# Six New 9,19-Cycloartane Triterpenoids from *Cimicifuga foetida* L.

**DOI:** 10.1007/s13659-016-0097-3

**Published:** 2016-05-20

**Authors:** Guo-Lei Zhu, Di-Fan Zhu, Luo-Sheng Wan, Xing-Rong Peng, Ni-Man Bao, Zhi-Run Zhang, Lin Zhou, Ming-Hua Qiu

**Affiliations:** State Key Laboratory of Phytochemistry and Plant Resources in West China, Kunming Institute of Botany, Chinese Academy of Sciences (CAS), Kunming, 650201 People’s Republic of China; Graduate University of Chinese Academy of Sciences, Beijing, 100049 People’s Republic of China; Faculty of Life Science and Technology, Kunming University of Science and Technology, Kunming, 650500 Yunnan People’s Republic of China

**Keywords:** *Cimicifuga foetida*, 9,19-Cycloartane triterpenoids, Cycloartane-type

## Abstract

Six new 9,19-cycloartane triterpene derivatives, as well as 3 known analogues (**7**–**9**), were isolated from the roots of *Cimicifuga foetida* L. Their structures were established on the basis of extensive spectroscopic analyses (IR, UV, ORD, HRESIMS, 1D and 2D NMR).

**Electronic supplementary material:**

The online version of this article (doi:10.1007/s13659-016-0097-3) contains supplementary material, which is available to authorized users.

## Introduction

*Cimicifuga foetida L*. or *actaea foetida* also named “Shengma”, a well known medicinal plant widely distributed in China, has been used for alleviation fever, pain, and inflammation since ancient times in China [1–3]. Currently, it is prescribed as one of the source plants for the treatment of headache, sore throat, toothache, and uterine prolapse in the *Chinese Pharmacopoeia* 2010 [4]. Phytochemical investigation have shown that 9,19-cycloartane triterpenoids and their glycosides are the main constituents of *Cimicifuga*. Meanwhile, because of their structural diversity and significant antitumor activity, this kind of components attracted so much attention [5–14].

In our continuous search for the bioactive triterpenoids, six new cycloartane-type triterpenoids, namely 4′-*O*-acetyl-cimigenol-3-*O*-*β*-d-xylopyranoside (**1**), 2′,12-*O*-diacetyl-25-anhydrocimicigenol-3-*O*-*β*-d-xylopyranoside (**2**), 12*β*-hydroxy-1,19:9,11-didehydro-9,10-*seco*-cimigenol-3-*O*-*β*-d-xylopy-ranoside (**3**), (23*S*, 24*R*)-12*β*-hydroxy-7,8-dihydro-12-deacetyl-acetaeaepoxide-3-one. (**4**), 16,17-dide- hydro-2′,24-*O*-diacetylhydroshengmanol-3-*O*-*β*-d-xylopyranoside (**5**), and (23*S*,24*S*,25*S*)-16,23:23,- 26-diepoxy-24,25-dihydroxy-9,19-cycloart-1,2-en-3,12-dione (**6**), together with three known analogues, asiaticoside A (**7**), 24(*S*)-*O*-acetylhydroshengmanol-3-*O*-*β*-d-xylopyranoside (**8**), and cimisterol A (**9**) were isolated from the roots of *C. foetida*. All the new compounds were evaluated for their cytotoxicities against five selected human cancer cell lines (HL-60, SMMC-7721, A-549, MCF-7 and SW480).

## Results and Discussion

**Fig. 1:**
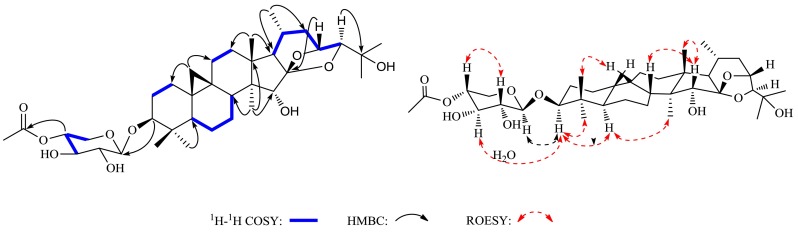
Major correlations in 2D NMR spectra of compound **1**

Compound **1** had the molecular formula of C_37_H_58_O_10_, which was determined by its HR-EIMS at *m/z* 662.4414 [M]^+^. The IR spectrum showed absorption for hydroxyl group at 3425 cm^−1^. The ^1^H NMR spectrum (Table 1) showed characteristic cyclopropane methylene signals at *δ*_H_ 0.29 and 0.52 (each 1H, d, *J* = 3.8 Hz), a secondary methyl at *δ*_H_ 0.86 (d, *J* = 6.5 Hz), six tertiary methyls at *δ*_H_ 1.01 to 1.52 (each 3H, s), an acetyl group at *δ*_H_ 1.98, and an anomeric proton at *δ*_H_ 4.89 (d, *J* = 7.6 Hz). The ^13^C NMR and DEPT spectroscopic data (Table 2) of **1** displayed a characteristic cimigenol-type triterpenoid carbon resonances, corresponding to the methylene carbon of the cyclopropane ring at *δ*_C_ 31.2 (C-19), four oxymethine carbons at *δ*_C_ 89.0 (C-3), 80.6 (C-15), 72.2 (C-23), and 90.5 (C-24), and two oxygened quaternary carbons at *δ*_C_ 112.3 and 71.3. Apart from above data, a glycosidic moiety signals [*δ*_C_ 107.7 (d), 76.1 (d), 75.3 (d), 73.5 (d) and 63.5 (t)] were also obseverd in its ^13^C NMR spectrum. These data showed similarities as those of cimigenol-3-*O*-[2′-*O*-acetyl]-*β*-d-xylopyranoside [12]. However, a detailed comparison of their 1D NMR spectra revealed that they had different sugar unit. The upfield shift of H-2′ (*δ*_H_ 5.56 → *δ*_H_ 4.08) and the downfield shift of H-4′ (*δ*_H_ 4.30 → *δ*_H_ 5.44) in their ^1^H NMR spectra, along with the HMBC correlation (Fig. 1) of H-4′ (*δ*_H_ 5.44) with the carbonyl group of the acetoxyl group (*δ*_C_ 171.0) and of the anomeric proton with C-3, indicated that a 4′-*O*-acetyl-xylopyranosyl at C-3 in **1** replaced the 2′-*O*-acetyl-xylopyranosyl in cimigenol-3-*O*-[2′-O-acetyl]-*β*-d-xylopyranoside. The sugar unit of **1** was further confirmed by comparing its TLC and specific rotation with a standard after acid hydrolysis. Thus, the planar structure of **1** was determined.

**Table 1:** ^1^H (600 MHz) NMR data of compounds **1**–**6** in Pyridine-*d*_5_ [*δ* in ppm, *J* in Hz]

No.	**1**	**2**	**3**	**4**	**5**	**6**
1	1.56 m 1.69 m	1.07 m 1.52 m	1.47 m 2.06 m	1.43 m 1.71 m	1.20 m 1.50 m	6.65 d (10.0)
2	1.95 dt (12.6, 3.7) 2.32 dt (12.6, 3.7)	1.26 m 1.82 dt (12.6, 3.7)	1.85 m 2.36 m	2.30 m 2.63 m	1.89 m 2.28 m	6.12 d (10.0)
3	3.51 dd (11.7, 4.3)	3.38 dd (11.7, 4.4)	3.57 dd (4.6, 11.5)	–	3.38 dd (11.7, 4.4)	–
5	1.30 m	1.27 m	2.04 m	1.57 dd (12.1, 4.2)	1.33 dd (13.0, 2.6)	1.89 dd (12.7, 5.2)
6	0.72 q (12.4) 1.51^a^	0.72 q (12.7) 1.53 m	1.30 m 2.68 m	0.99 m 1.37 m	0.76 q (13.0) 1.55 m	1.32 m 2.32 d (19.7)
7	1.07^a^ 2.10 m	1.08 m 2.23 m	2.19 dd (12.4, 4.5) 2.25 dd (18.1, 4.5)	0.96 m 1.30 m	1.19 m 1.55 m	0.85 m 1.41 m
8	1.69 m	1.73 dd (12.5, 4.4)	2.56 d (10.9)	1.66 dd (12.0, 5.4)	1.88 m	2.32 m
11	1.16^a^ 2.11 m	1.13 m 2.94 dd (16.1, 9.5)	5.92 br s	1.49 m 2.64 m	1.05 m 1.13 m	2.33 s 2.92 d (19.9)
12	1.69 m 1.56 m	5.27 dd (9.3, 2.50)	4.54 m	4.10 m	1.56 m 1.90 m	–
15	4.29 br s	4.47 d (8.8)	4.59 d (6.4)	1.84 m 1.96 m	4.75 d (2.9)	1.88 dd (12.7, 5.2) 2.32 m
16	–	–	–	5.09 t (8.2)	–	4.80 q (7.5 Hz)
17	1.52^a^	1.64^a^	1.86 d (12.2)	1.95 m	–	2.33 m
18	1.16 s	1.33 s	1.16 s	1.51 s	1.27 s	1.38 s
19	0.29 d (3.8) 0.52 d (3.8)	0.26 d (4.2) 0.53 d (4.2)	6.11 br s	0.48 d (4.3) 0.70 d (4.3)	0.17 d (3.8) 0.56 d (3.8)	0.87 d (4.8) 1.14 d (4.8)
20	1.69 m	1.64 br s	1.79 m	2.41 m	2.56 m	2.33 m
21	0.86 d (6.5)	0.94 d (6.0)	1.42 d (6.4)	1.80 d (6.1)	1.00 d (6.7)	1.32 d (6.2)
22	1.03 m 2.27 dt (7.1, 2.2)	1.00 s 2.24 m	1.13 m 2.39 m	4.02 d (10.6)	1.85 m 2.05 m	2.08 m
23	4.79 d (9.0)	4.31 m	4.80 d (8.7)	–	4.60 d (11.8)	–
24	3.80 br s	4.19 br s	3.85 s	4.26 br s	5.43 d (2.5)	4.53 d (6.6)
26	1.52 s	5.39 s 4.90 s	1.53 s	1.72 s	1.60 s	4.31 d (8.8) 4.38 d (8.8)
27	1.32 s	1.86 s	1.54 s	1.80 s	1.63 s	1.97 s
28	1.01 s	1.33 s	1.15 s	0.85 s	0.99 s	0.67 s
29	1.16 s	1.10 s	1.34 s	1.23 s	1.04 s	1.23 s
30	1.06 s	0.96 s	0.95 s	0.97 s	1.10 q	0.97 s
AcO-12		2.15 s				
AcO-24					2.09 s	
Sugar						
1′	4.89 d (7.6)	4.83 d (8.0)	4.85 d (7.6)		4.86 d (8.0 Hz)	
2′	4.08 t (8.0)	5.59 t (8.0)	4.03 m		5.60 t (8.0 Hz)	
3′	4.31 t (8.0)	4.19 m	4.19 t (8.8)		4.21 m	
4′	5.44 dt (11.3, 5.4)	4.21 m	4.25 m		4.25 dd (13.9, 5.1)	
5′	3.62 t (10.7) 4.32 dd (11.3, 5.4)	3.68 t (11.1) 4.31 m	3.76 t (8.8) 4.39 t (8.8)		3.75 t (11.1 Hz) 4.36 dd (11.1, 5.0)	
AcO-2′		2.14 s			2.17 s	
AcO-4′	1.98 s					

^a^Signals overlapped

**Table 2:** ^13^C (150 MHz) NMR data of compounds **1**–**6** [*δ* in ppm, *J* in Hz]

No.	**1**	**2**	**3**	**4**	**5**	**6**
1	32.7 t	32.5 t	40.6 t	33.7 t	32.0 t	153.5 d
2	30.4 t	30.3 t	32.6 t	37.8 t	30.2 t	127.2 d
3	89.0 d	88.7 d	87.8 d	215.3 s	88.9 d	204.3 s
4	41.7 s	41.3 s	42.7 s	50.4 s	41.3 s	46.6 s
5	47.9 d	47.3 d	51.1 d	48.4 d	47.8 d	47.2 d
6	21.4 t	21.1 t	25.0 t	21.4 t	21.3 t	25.7 t
7	26.7 t	26.3 t	30.6 t	26.1 t	30.3 t	20.3 t
8	49.0 d	47.6 d	50.4 d	46.1 d	47.1 d	45.7 d
9	20.3 s	20.5 s	140.3 s	21.9 s	20.2 s	25.8 s
10	26.9 s	27.0 s	138.7 s	26.6 s	27.4 s	30.6 s
11	26.7 t	37.8 t	135.9 d	41.0 t	26.7 t	46.1 t
12	34.4 t	77.6 d	46.7 s	72.6 d	30.3 t	210.8 s
13	42.2 s	46.3 s	46.7 s	51.5 s	47.5 s	47.5 s
14	47.6 s	48.6 s	49.8 s	48.2 s	49.9 s	60.8 s
15	80.6 d	79.6 d	79.2 d	44.0 t	79.3 d	46.1 t
16	112.3 s	112.6 s	112.7 s	72.8 d	151.3 s	73.0 d
17	59.9 d	59.9 d	58.7 d	53.3 d	121.7 s	48.7 d
18	19.0 q	13.1 q	11.9 q	13.7 q	24.6 q	14.6 q
19	31.2 t	31.2 t	129.6 d	29.6 t	32.4 t	32.6 t
20	24.4 d	24.3 d	24.0 d	35.1 d	27.9 d	25.7 d
21	19.9 q	20.1 q	21.2 q	18.8 q	20.7 q	21.8 q
22	38.5 t	38.8 t	38.9 t	87.9 d	37.2 t	41.3 t
23	72.2 d	74.9 d	72.4 d	106.0 s	77.0 d	106.9 s
24	90.5 d	86.9 d	90.6 d	83.8 d	80.2 d	86.3 d
25	71.3 s	146.1 s	71.4 s	83.9 s	72.3 s	78.2 s
26	24.3 q	113.5 t	27.6 q	25.3 q	27.1 q	78.9 t
27	25.7 q	18.5 q	26.0 q	28.3 q	28.5 q	23.6 q
28	12.2 q	12.4 q	10.4 q	21.1 q	15.6 q	20.3 q
29	26.0 q	25.7 q	25.0 q	22.9 q	25.7 q	22.2 q
30	15.7 q	15.5 q	15.4 q	20.1 q	14.6 q	19.6 q
AcO-12		21.6 q				
		170.9 s				
AcO-24					21.3 q	
					171.4 s	
Sugar						
1′	107.7 d	105.0 d	108.0 d		105.0 d	
2′	76.1 d	76.0 d	76.1 d		76.0 d	
3′	75.3 d	76.6 d	79.2 d		76.6 d	
4′	73.5 d	71.9 d	71.7 d		71.7 d	
5′	63.5 t	67.4 t	67.7 t		67.5 t	
AcO-2′		170.4 1			170.4 s	
		22.0			21.6 q	
AcO-4′	21.2 q					
	171.0 s

In the ROESY spectrum (Fig. 1), correlations of H-3/H-5 and H-15/H_3_-18 suggested that H-3 and H-15 were *α*- and *β*-oriented, respectively. Moreover, the configurations of C-23 and C-24 were assigned as *R* and *S*, respectively, by comparing the coupling constants of H-23 (9.0 Hz) and H-24 (0 Hz) of **1** with those of known compounds [15]. Therefore, compound **1** was established to be 4′-*O*-acetylcimigenol-3-*O*-*β*-d-xylopyranoside.

Compound **2** was obtained as a white powder and gave a molecular formula of C_39_H_58_O_11_ by its HR-EIMS (*m/z* 702.3974 [M]^+^). The ^1^H and ^13^C NMR spectra (Tables 1, 2) of **2** were very similar to those of 25-anhydrocimicigenol-3-*O*-[2′-*O*-acetyl]-*β*-d-xylopyranoside [16], with the exception of an additional acetyl group, which was assigned to C-12 on the basis of the HMBC correlation of H-12 (*δ*_H_ 5.27) with the acetyl carbonyl carbon at *δ*_C_ 170.9 and the ^1^H-^1^H COSY cross peak of H-12 (*δ*_H_ 1.13) with H-11 (*δ*_H_ 2.94). Significant ROESY correlation of H-12 with H-17 indicated a *β*-orientation of the substituent acetyl group at C-12. Therefore, the structure of **2** was determined as 2′,12-*O*-diacetyl-25-anhydrocimicigenol-3-*O*-*β*-d-xylopyranoside.

**Fig. 2:**
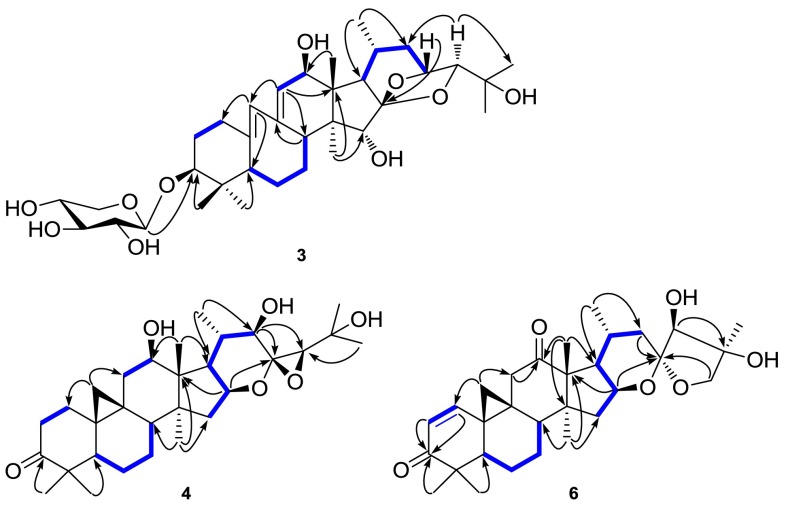
Key HMBC () and ^1^H-^1^H COSY () correlations of compounds **3, 4** and **6**

Compound **3** was isolated as a white powder, showing [M + Na]^+^ ion at *m/z* 657.3602 in the HR-ESIMS consistent with the empirical molecular formula C_35_H_54_O_10_ (calcd 657.3615), requiring 9 sites of unsaturation. The IR and UV spectra exhibited absorption bands for hydroxyl group (3431 cm^−1^) and conjugated double bond (λ_max _249 nm; 1631 cm^−1^), respectively. The ^1^H NMR spectrum exhibited a *sec*-methyl signal at *δ*_H_ 1.42 (3H, d, *J* = 6.4 Hz), six *tert*-methyls at *δ*_H_ 0.95–1.54 (each 3H, s), two olefinic methine signals at *δ*_H_ 5.92 (1H, br s) and 6.11 (1H, br s), and an anomeric methine signal at *δ*_H_ 4.85 (1H, d, *J* = 7.6 Hz). The ^13^C NMR showed 35 carbon resonances (Table 2), of which 30 were attributed to a triterpene skeleton and five to a pentose. A DEPT NMR experiment permitted differentiation of the 30 carbon signals into seven methyls, five methylenes, eleven methines (including five oxygenated and two olefinic signals), and seven quaternary carbons (including two oxygenated and two olefinic signals). The diagnostic signals of two oxygen-bearing methine carbons at *δ*_C_ 90.6 (C-24) and 70.4 (C-23), and a ketal carbon at *δ*_C_ at 112.7 suggested that **3** was a cimigenol-type triterpene compound. Further inspection of the 1D NMR and HSQC spectra of **3**, the characteristic cyclopropane methylene resonances H_2_-19 and two quaternary carbons (C-9 and C-10) were not observed at the characteristic high magnetic field. Besides, comparison the NMR spectra of **3** with those of 12*β*-hydroxycimigenol-3-*O*-*β*-d-xylopyranoside [17], the signals due to C-9, C-10, C-11, and C-19 showed a downfield shift from *δ*_C_ 20.5, 26.1, 40.6, and 30.8 to 140.3, 138.7, 135.9, and 129.6, respectively, in **3**. Such evidences indicated that **3** was a 9,10-*seco*-9,19-cyclolanostane glycoside with two double bonds. And the location of the double bonds (C_10_=C19 and C_9_=C_11_) could be further deduced. This was further supported by IR, UV and 2D NMR spectra (Fig. 2). Furthermore, the configurations of C-23 and C-24 were assigned as *R* and *S*, respectively, by the same way as **1**. Ultimately, the structure of **3** was determined as 12*β*-hydroxy-10,19:9,11-didehydro-9,10-*seco*-cimigenol-3-*O*-*β*-d-xylopyranoside.

The molecular of compound **4** was assigned as C_30_H_46_O_6_ by HR-EIMS at *m/z* 502.3294 [M]^+^. The 1D NMR data of **4** (Tables 1, 2) showed that **4** was a highly oxygenated 9,19-cycloartane triterpene and resembled that of the aglycone of 7,8-dihydroactaeaepoxide-3-*O*-*β*-d-xylopyranoside [18]. However, the signals for the oxymethine at C-3 and the acetoxyl group at C-12 were absent. Instead a carbonyl group signal at *δ*_C_ 215.3 and an upfield oxymethine at *δ*_C_ 72.6 were observed, which indicated that the oxymethine (C-3) and the acetoxyl group (C-12) were replaced by a carbonyl group and a hydroxyl group, respectively. The evidence was established from HMBC correlations (Fig. 2) of H_3_-29 (*δ*_H_ 1.23) and H_3_-30 (*δ*_H_ 0.94) with C-3 (*δ*_C_ 215.3), of H_3_-18 (*δ*_H_ 1.51) with C-12 (*δ*_C_ 72.6), together with the ^1^H-^1^H COSY correlations (Fig. 2) of H-1 (*δ*_H_ 1.43, 1.71)/H-2 (*δ*_H_ 2.30, 2.63), H-5 (*δ*_H_ 1.57)/H-6 (*δ*_H_ 0.99, 1.37)/H-7 (*δ*_H_ 0.96, 1.30)/H-8 (*δ*_H_ 1.66), H-11 (*δ*_H_ 1.49, 2.64)/H-12 (*δ*_H_ 4.10), H-15 (*δ*_H_ 1.84, 1.96)/H-16(*δ*_H_ 5.09)/H-17 (*δ*_H_ 1.53). Thus, the planar structure of **4** was elucidated. Additionally, the substituent hydroxyl group at C-12 was assigned as *β* orientation by the ROESY correlation of H_3_-28 with H-12. Further inspection of the ROESY spectrum, the correlations of H-16 with H-22 and H-24, of H-22 with H-12, H-21, and H-28, and of H-24 with H-16, suggested that an *α* orientation of H-16, H-22, H-12, and H-24, respectively. So the configuration of C-23 was elucidated as *S* since C-23 and C-24 formed an oxirane. Ultimately, compound **4** was elucidated as (23*S*, 24*R*)-12*β*-hydroxy-7,8-dihydro-12-deacetylacetaeaepoxide-3-one.

Compound **5** was determined to have the molecular formula of C_39_H_60_O_11_ by its HR-EIMS (*m/z* 704.4138 [M]^+^). The ^1^H NMR spectrum (Table 1) displayed characteristic cyclopropane methylene signals at *δ*_H_ 0.17 and 0.56 (each 1H, d, *J* = 3.8 Hz), a *sec*-methyl at *δ*_H_ 1.00 (3H, d, *J* = 6.7 Hz), six *tert*-methyls at *δ*_H_ 0.99–1.63 (each 3H, s), an acetyl methyl group at *δ*_H_ 2.09 (3H, s), and an anomeric proton signal at *δ*_H_ 4.86 (1H, d, *J* = 8.0 Hz), respectively, suggersting **5** to be a 9,19-cycloartane triterpene glycoside with a substituent acetyl group. The NMR data (Tables 1, 2) of **5** resembled those of 25-*O*-methyl-24-*O*-acetylhydroshengmanol-3-*O*-*β*-d-xylopyranoside [19], except for one more acetyl group for the sugar unit, one less substituent methoxy group at C-25, and the presence of two downfield signals at *δ*_C_ 151.3 and 121.7 while the absence of an oxygen-bearing quaternary carbon and a methine resonance due to C-16 and C-17, respectively. On the basis of these observations, it was reasonable to deduce that **5** was a 16,17-dehydrated derivative with an additional acetyl group and a skimpy methoxy group of 25-*O*-methyl-24-*O*-acetylhydroshengmanol-3-*O*-*β*-d-xylopyranoside. As for **2**, an acetyl group was determined to be at C-2′ for **5**, which was further confirmed by the HMBC correlation between the H-2′ signal at *δ*_H_ 5.20 and the carbonyl group signal at *δ*_C_ 170.4. In the NMR spectra, the signals of the methoxy appeared in 25-*O*-methyl-24-*O*-acetylhydro-shengmanol-3-*O*-*β*-d-xylopyranoside couldn’t be observed in **5**, while the chemical shift of C-25 shifted upfield from *δ*_C_ 76.0 to 72.3. This suggested the methoxy group located at C-25 wasn’t appeared in **5**. Additionally, the location of the double bond was further confirmed by the HMBC correlations of H-18 (*δ*_H_ 1.27) and H-21 (*δ*_H_ 1.00) with *δ*_C_ 121.7, of H-15 (*δ*_H_ 4.75) with *δ*_C_ 151.3 and 121.7, and of H-23 (*δ*_H_ 4.60) with *δ*_C_ 151.3, respectively. The configuration of C-23 could be determined as *β* by ROESY correlation of H-23 with H-20. And the configuration of C-24 was assigned as *S* by comparison the coupling constant of H-24 (*J* = 2.5 Hz) with those of dahurinyl deacetate (*J* = 9 Hz, 24*R*) and isodahurinyl deacetate (*J* = 2.0 Hz, 24*S*) [20]. Therefore, **5** was elucidated as 16,17-didehydro-2′,24-*O*-diacetyl- hydroshengmanol-3-*O*-*β*-d-xylopyranoside.

Compound **6** was isolated as a white powder. Its molecular formula (C_30_H_42_O_6_) was deduced from HR-EIMS (*m/z* 498.2991 [M]^+^), corresponding to nine degrees of unsaturation. The ^1^H and ^13^C NMR spectroscopic data (Tables 1, 2) of **6** showed similarities with those of yunnanterpene A [21], except for the differences of rings A and C, and the chemical shifts of C-22, C-23, and C-24. Two methylene signals due to C-1 at *δ*_C_ 33.3 and C-2 at *δ*_C_ 37.4 appeared in the ring A of yunnanterpene A were absent from the ^13^C-DEPT spectrum of **6**, respectively. Instead, two olefinic carbon signals at *δ*_C_ 153.5 and 127.2 were observed. Besides, the signal due to C-3 showed an upfield shift from *δ*_C_ 215.0 to 203.4. These evidences suggested the double bond was located at C-1 and C-2, which was further confirmed by the UV, IR (λ_max_ 262 nm; 1669 cm^−1^), and the HMBC correlations (Fig. 2) of the olefinic protons at *δ*_H_ 6.65 and 6.12 with the carbonyl carbon signals at *δ*_C_ 204.3 (C-3), The other changes of the ring C was that the methine signal at *δ*_C_ 72.1 (C-12) appeared in yunnanterpene A was absent instead of a quarternary carbonyl carbon signal at *δ*_C_ 210.8 (C-12) in **6**. Meanwhile, the ^13^C NMR signal due to C-11 showed a downfield shift from *δ*_C_ 40.6 in yunnanterpene A to 46.1 in 6, and the signal due to C-13 exhibited an unfield from *δ*_C_ 50.5 to 46.6, respectively. These observations indicated that the hydroxyl group was attached to C-12 was replaced by the carbonyl group, which was further confirmed by the HMBC correlations (Fig. 2) from H-11 (*δ*_H_ 2.33 and 2.92) and H_3_-18 (*δ*_H_ 1.38) to C-12 (*δ*_C_ 210.8), respectively. All of the above observations were consistent with the HSQC, HMBC, and ^1^H-^1^H COSY correlations (Fig. 2). Besides, the downfield shift of C-22 and C-24 from *δ*_C_ 37.5 and 83.7 to 41.3 and 86.3 and the upfield of C-23 from *δ*_C_ 110.9 to 106.9 suggested the configurations of two compounds may be different. Furthermore, the diagnostic ROESY configurations of H-16 with H-16, H_3_-28 and H_3_-27, of H-24 with H-26*α*, of H_3_-27 with H-16 and H-26*α*, indicated that the *α*-orientation of H-16, H_3_-27, and H-24. And the configuration of C-23 was identified as *S* by comparision the chemical shifts of C-16 (*δ*_C_ 73.0) and C-20 (*δ*_C_ 25.7) with the 26-deoxyactein compound [C-16 (*δ*_C_ 73.0) and C-20 (*δ*_C_ 26.0)] [16] and 23-*epi*-26-deoxyactein [C-16 (*δ*_C_ 74.5) and C-20 (*δ*_C_ 23.0)] [22]. Hence, compound **6** was determined as (23*S*,24*S*,25*S*)-16,23:23,26-diepoxy-24,25-dihydroxy-9,19-cycloart-1,2-en-3,12-dione.

Three known compounds asiaticoside A (**7**) [23], 24(*S*)-*O*-acetylhydroshengmanol-3-*O*-*β*-d-xylopyranoside (**8**) [19], and cimisterol A (**9**) [13] were also isolated from this species. Their structures were identified by its 1D NMR spectra as well as comparison with reported data.

Compounds **1**–**6** isolated in the present study were evaluated for their cytotoxicities against five human cancer cell lines using MTT method, with cisplatin and taxol as the positive control. Unfortunately, none of them showed significant activity [24].

## Experiments Section

### General Experimental Procedures

Optical rotations were measured in MeOH with a Horiba SEAP-300 polarimeter. ^1^H and ^13^C NMR spectra were recorded in pyridine-*d*_5_ on Bruker Avance III-600 MHz spectrometers (Bruker, Zürich, Switzerland), using TMS as internal standard for chemical shifts. Chemical shifts (*δ*) were expressed in ppm with reference to the TMS resonance. ESIMS, HRTOF-ESIMS and EIMS, HR-EIMS data were obtained using a VG Autospec-3000 and API QSTAR TOF spectrometer, respectively. Infrared spectra were recorded on a Shimadzu IR-450 instrument with KBr pellets. CD was detected with a Chirascan circular dichroism spectrograph (Applied Photophysis, England). Thin-layer chromatography was performed on precoated TLC plates (Silica gel GF254, Qingdao Marine Chemical, Inc.) and spots were visualized by heating after spraying with 10 % H_2_SO_4_ in EtOH. Semipreparative HPLC was performed on an Agilent 1100 liquid chromatograph with a YMC-Pack Pro C18 RS 10 mm × 250 mm column. Silica gel (mesh 200–300, Qingdao Marine Chemical, Inc.), Lichroprep RP-18 (40–63 µm, Merck), Amberlite IR-35 (10 mL) column and Sephadex LH-20 (Pharmacia) were used for column chromatography.

### Plant Materials

The roots of *Cimicifuga foetida* (82 kg) were collected from Yulong County, Yunnan Province, in September 2010 and identified by Professor Pei shengji, Kunming Institute of Botany, Chinese Academy of Sciences. A voucher specimen (KUN No. 20100906) has been deposited at the State Key Laboratory of Phytochemistry and Plant Resources in West China, Kunming Institute of Botany, Chinese Academy of Sciences, PR China.

### Extraction and Isolation

The air-dried roots of *C. foetida* (82 kg) were crushed with a blender and refluxed with 95 % EtOH at 70 °C for three times (5 h, each). The residue was yielded by removal of the solvent was dissolved in water to form a suspension. The aqueous suspension was successively partitioned with EtOAc and *n*-BuOH. The EtOAc (5.6 kg) fraction was absorbed on 12 kg silica gel and chromatographed on a prepacked (120 kg) silica gel column, eluting stepwise with CHCl_3_–MeOH (1:0, 100:1, 50:1, 20:1, 5:1) to give five fractions (A–E) Fraction C (230 g) was subjected to column chromatograph (CC) on silica gel (1.5 kg) and eluted with PE-Me_2_CO (5:1, 2:1, 0:1) to obtain C-1 (60 g), C-2 (40 g), and C-3 (105 g) as in the previous report [25]. Fraction C-2 (40 g) was purified using an ODS silica gel column with MeOH-H_2_O (60:40, 80:20, 100:0), followed by purification using preparative HPLC eluted with CH_3_CN-H_2_O (65:35), furnished compound **4** (2.3 mg). Similarly, using CH_3_CN-H_2_O (70:30) as eluent with a flow rate of 3 ml/min, compound **6** (9.6 mg) was purified from C-3 (105 g). Fraction D (200 g) was separated on silica gel eluted with CHCl_3_–Me_2_CO (gradient polarity from 15:1 to 5:1) to give ten subfractions (D-1-D-10). Fraction D-1 (10 g) was separated by CC (ODS silica gel) with MeOH–H_2_O (gradient polarity from 60:40 to 90:10) and purified by HPLC eluting with CH_3_CN–H_2_O (60:40, flow rate of 3 ml/min) to obtain **3** (6.0 mg) and **9** (2.5 mg) with retention times of 9.30 and 18.50 min, respectively. Sub-fraction D-6 (15 g) was subjected to silica gel (CH_3_Cl_3_–MeOH, gradient from 25:1 to 15:1) and then purified by an ODS silica gel column (MeOH–H_2_O, 70:30 to 100:0) and HPLC spectrum eluting with (CH_3_CN–H_2_O, 50:50, flow rate of 3 ml/min) to obtain **1** (6.2 mg) and **5** (5.1 mg) with retention times of 10.80 and 13.40 min, respectively. Sub-fraction D-8 (20 g) was chromatographed on a silica gel (CH_3_Cl_3_–MeOH, 30:1, 20:1, 15:1) and ODS (MeOH–H_2_O, 70:30 to 100:1), followed purified on HPLC CH_3_CN–H_2_O (67:33, flow rate of 3 ml/min) to yield **2** (5.3 mg), **7** (5.5 mg), and **8** (6.0 mg) with the retention times of 19.20, 26.70, and 12.40 min, respectively.

### 4′-*O*-Acetylcimigenol-3-*O*-*β*-d-xylopyranoside (**1**)

White powder; $[\alpha ]_{\text{D}}^{20}$ −3.59 (*c* 0.2, MeOH); IR (KBr) *ν*_max_ 3425, 2964, 2936, 2870, 1742, 1626, 1458, 1413, 1379, 1308, 1252, 1170, 1047, 979 cm^−1^; ^1^H and ^13^C NMR data see Tables 1, 2; HREIMS *m/z* 662.4414 [M]^+^ (calcd for 662.4394).

### 2′,12-*O*-Diacetyl-25-anhydrocimicigenol-3-*O*-*β*-d-xylopyranoside (**2**)

White powder; $[\alpha ]_{\text{D}}^{20}$ −1.63 (*c* 1.8, MeOH); IR (KBr) *ν*_max_ 3442, 2935, 2871, 1736, 1629, 1455, 1413, 1239, 1159, 1071, 1044, 982 cm^−1^; ^1^H and ^13^C NMR data see Tables 1, 2; HREIMS *m/z* 702.3974 [M]^+^ (calcd for 702.3979).

### 12*β*-Hydroxy-1,19:9,11-didehydro-9,10-*seco*-cimigenol-3-*O*-*β*-d-xylopyranoside (**3**)

White powder; $[\alpha ]_{\text{D}}^{20}$ −1.63 (*c* 0.01, MeOH); IR (KBr) *ν*_max_ 3431, 2931, 2873, 1631, 1456, 1384, 1238, 1161, 1041, 975 cm^−1^; ^1^H (C_5_D_5_N, 600 MHz) and ^13^C NMR (C_5_D_5_N, 150 MHz) data see Tables 1, 2; ESIMS *m/z* 657 [M + Na]^+^; HRESIMS *m/z* 657.3602 (calcd for 657.3615).

### (23*S*,24*R*)-12*β*-Hydroxy-7,8-dihydro-12-deacetyl- acetaeaepoxide-3-one. (**4**)

White powder; $[\alpha ]_{\text{D}}^{20}$ −18.17 (*c* 1.1, MeOH); IR (KBr) *ν*_max_ 3441, 2966, 2932, 2875, 1705, 1628, 1465, 1383, 1248, 1061 cm^−1^; ^1^H and ^13^C NMR data see Tables 1, 2; HREIMS *m/z* 502.3294 [M]^+^ (calcd for 502.3294).

### 16,17-Didehydro-2′,24-*O*-diacetylhydroshengmanol-3-*O*-*β*-d-xylopyranoside (**5**)

White powder; $[\alpha ]_{\text{D}}^{20}$ −10.28 (*c* 0.01, MeOH); IR (KBr) *ν*_max_ 3431, 2929, 2870, 2853, 1739, 1629, 1459, 1375, 1237, 1240, 1167, 1125, 1072, 1046, 980 cm^−1^; ^1^H and ^13^C NMR data see Tables 1, 2; HREIMS *m/z* 704.4138 [M]^+^ (calcd for 704.4136).

### (23*S*,24*S*,25*S*)-16,23:23,26-Diepoxy-24,25-diihydroxy-9,19-cycloart-1,2-en-3,12-dione (**6**)

White powder; $[\alpha ]_{\text{D}}^{20}$ −24.21 (*c* 0.5, MeOH); IR (KBr) *ν*_max_ 3440, 2931, 2871, 1712, 1669, 1457, 1380, 1278, 1167, 1019, 940 cm^−1^; ^1^H and ^13^C NMR data see Tables 1, 2; HRESIMS *m/z* 498.2991 [M]^+^ (calcd for 498.2981).

### Acidic Hydrolysis of **1**, **2** and **5**

The new compounds **1, 2** and **5** (4 mg of each) were dissolved in MeOH (5 mL) and refluxed with 0.5 N HCl (3 mL) for 4 h. Each reaction mixture was diluted with H_2_O and extracted with CHCl_3_ (3 × 10 mL). The water layer was then neutralized by Ag_2_CO_3_, and the precipitate filtered to give a monosaccharide. Each monosaccharide of those compounds had an *R*_*f*_ (EtOAc/CHCl_3_/MeOH/H_2_O, 3:2:2:1) and specific rotation $[\alpha ]_{\text{D}}^{20}$ +24.3 (*c* 0.10, H_2_O) corresponding to those of d-xylose (Sigma-Aldrich).

## Electronic supplementary material

Below is the link to the electronic supplementary material.

Supplementary material 1 (DOC 3562 kb)
