# Supplementary material for: Six New 9,19-Cycloartane Triterpenoids from Cimicifuga foetida L
Source: Nat Prod Bioprospect. 2016 May 20;6(4):187–93. doi: 10.1007/s13659-016-0097-3 (PMC4940256; doi:10.1007/s13659-016-0097-3)

**Supplementary data**

**Six new 9,19-cycloartane tripenoids from *Cimicifuga foetida* L.**

Guo-Lei Zhu,^a,b^ Yin Nian,^a^ Di-Fan Zhu,^c^ Luo-Sheng Wan,^a^ Ni-Man Bao,^a^ Wei-Hua Wang,^a^ Lin Zhou,^a^ Ming-Hua Qiu**^*^**

^a^State Key Laboratory of Phytochemistry and Plant Resources in West China, Kunming Institute of Botany, Chinese Academy of Sciences (CAS), Kunming 650204, PR China.

^b^Graduate University of Chinese Academy of Sciences, Beijing 100049, PR China.

^c^Faculty of Life Science and Technology, Kunming University of Science and Technology, Kuming 650500, Yunnan, PR China.

**Corresponding Authors**

* Ming-Hua Qiu

*E-mail*: mhchiu@mail.kib.ac.cn (M.H. Qiu).

Tel/Fax: +86-25-65223327.

**Contents**

[Figure S1. ^1^H NMR spectrum of compound 1 (Pyridine *d*_5_, 600 MHz). 1](#__RefHeading___Toc443493166)

[Figure S2. ^13^C NMR spectrum of compound 1 (Pyridine *d*_5_, 150Hz). 1](#__RefHeading___Toc443493167)

[Figure S3. HSQC spectrum of compound 1 (Pyridine *d*_5_). 2](#__RefHeading___Toc443493168)

[Figure S4. HMBC spectrum of compound 1 (Pyridine *d*_5_). 2](#__RefHeading___Toc443493169)

[Figure S5. COSY spectrum of compound 1 (Pyridine *d*_5_) 3](#__RefHeading___Toc443493170)

[Figure S6 ROESY spectrum of compound 1 (Pyridine *d*_5_). 3](#__RefHeading___Toc443493171)

[Figure S7 HREIMS spectrum of compound 1. 4](#__RefHeading___Toc443493172)

[Figure S8. ^1^H NMR spectrum of compound 2 (Pyridine *d*_5_, 600 MHz) 4](#__RefHeading___Toc443493173)

[Figure S9. ^13^C NMR spectrum of compound 2 (Pyridine *d*_5_, 150Hz). 5](#__RefHeading___Toc443493174)

[Figure S10. HSQC spectrum of compound 2 (Pyridine *d*_5_). 5](#__RefHeading___Toc443493175)

[Figure S11. HMBC spectrum of compound 2 (Pyridine *d*_5_). 6](#__RefHeading___Toc443493176)

[Figure S12. COSY spectrum of compound 2 (Pyridine *d*_5_) 6](#__RefHeading___Toc443493177)

[Figure S13. ROESY spectrum of compound 2 (Pyridine *d*_5_). 7](#__RefHeading___Toc443493178)

[Figure S14. HREIMS spectrum of compound 2. 7](#__RefHeading___Toc443493179)

[Figure S15. ^1^H NMR spectrum of compound 3 (Pyridine *d*_5_, 600 MHz) 8](#__RefHeading___Toc443493180)

[Figure S16. ^13^C NMR spectrum of compound 3 (Pyridine *d*_5_, 150Hz). 8](#__RefHeading___Toc443493181)

[Figure S17. HSQC spectrum of compound 3 (Pyridine *d*_5_). 9](#__RefHeading___Toc443493182)

[Figure S18. HMBC spectrum of compound 3 (Pyridine *d*_5_). 9](#__RefHeading___Toc443493183)

[Figure S19. COSY spectrum of compound 3 (Pyridine *d*_5_) 10](#__RefHeading___Toc443493184)

[Figure S20. HREIMS spectrum of compound 3. 10](#__RefHeading___Toc443493185)

[Figure S21.^1^H NMR spectrum of compound 4 (Pyridine *d*_5_, 600 MHz). 11](#__RefHeading___Toc443493186)

[Figure S22.^13^C NMR spectrum of compound 4 (Pyridine *d*_5_, 150Hz). 11](#__RefHeading___Toc443493187)

[Figure S23. HSQC spectrum of compound 4 (Pyridine *d*_5_). 12](#__RefHeading___Toc443493188)

[Figure S24. HMBC spectrum of compound 4 (Pyridine *d*_5_). 12](#__RefHeading___Toc443493189)

[Figure S25. COSY spectrum of compound 4 (Pyridine *d*_5_). 13](#__RefHeading___Toc443493190)

[Figure S26. ROESY spectrum of compound 4 (Pyridine *d*_5_). 13](#__RefHeading___Toc443493191)

[Figure S27. HREIMS spectrum of compound 4. 14](#__RefHeading___Toc443493192)

[Figure S28. ^1^H NMR spectrum of compound 5 (Pyridine *d*_5_, 600 MHz) 14](#__RefHeading___Toc443493193)

[Figure S29. ^13^C NMR spectrum of compound 5 (Pyridine *d*_5_, 150Hz). 15](#__RefHeading___Toc443493194)

[Figure S30. HSQC spectrum of compound 5 (Pyridine *d*_5_). 15](#__RefHeading___Toc443493195)

[Figure S31. HMBC spectrum of compound 5 (Pyridine *d*_5_). 16](#__RefHeading___Toc443493196)

[Figure S32. COSY spectrum of compound 5 (Pyridine *d*_5_) 16](#__RefHeading___Toc443493197)

[Figure S33. ROESY spectrum of compound 5 (Pyridine *d*_5_). 17](#__RefHeading___Toc443493198)

[Figure S34. HREIMS spectrum of compound 5. 17](#__RefHeading___Toc443493199)

[Figure S35. ^1^H NMR spectrum of compound 6 (Pyridine *d*_5_, 600 MHz) 18](#__RefHeading___Toc443493200)

[Figure S36. ^13^C NMR spectrum of compound 6 (Pyridine *d*_5_, 150Hz). 18](#__RefHeading___Toc443493201)

[Figure S37. HSQC spectrum of compound 6 (Pyridine *d*_5_). 19](#__RefHeading___Toc443493202)

[Figure S38. HMBC spectrum of compound 6 (Pyridine d_5_). 19](#__RefHeading___Toc443493203)

[Figure S39. COSY spectrum of compound 6 (Pyridine *d*_5_) 20](#__RefHeading___Toc443493204)

[Figure S40. ROESY spectrum of compound 6 (Pyridine *d*_5_). 20](#__RefHeading___Toc443493205)

[Figure S41. HREIMS spectrum of compound 6. 21](#__RefHeading___Toc443493206)

# Figure S1. ^1^H NMR spectrum of compound 1 (Pyridine *d*_5_, 600 MHz)
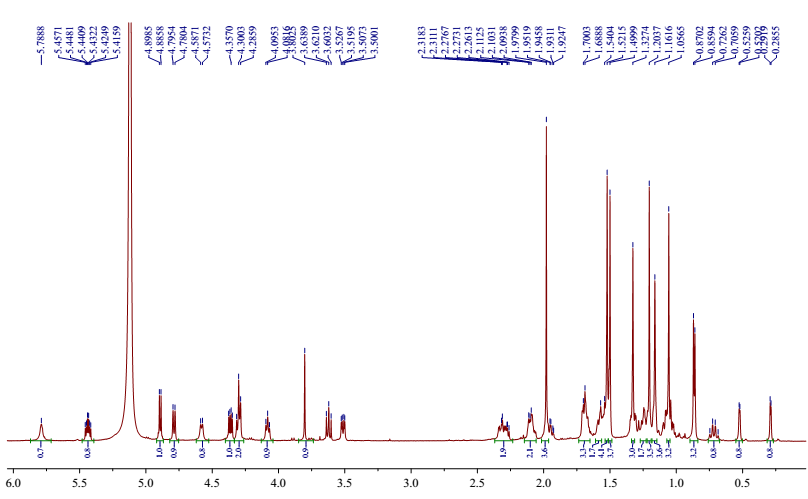


# Figure S2. ^13^C NMR spectrum of compound 1 (Pyridine *d*_5_, 150Hz)

**
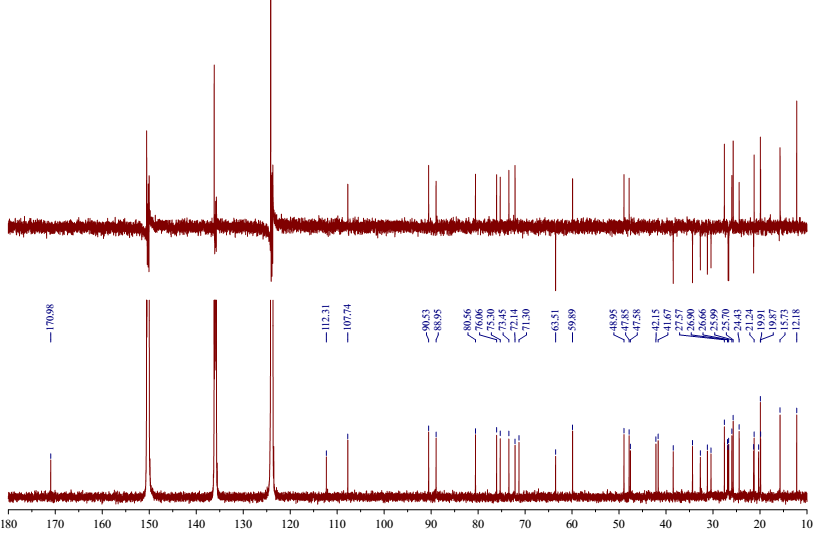
**

# Figure S3. HSQC spectrum of compound 1 (Pyridine *d*_5_)
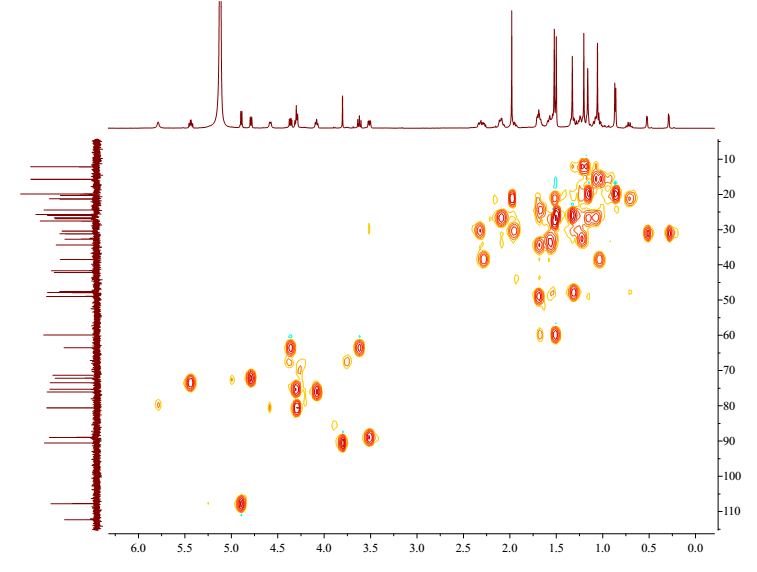


# Figure S4. HMBC spectrum of compound 1 (Pyridine *d*_5_)
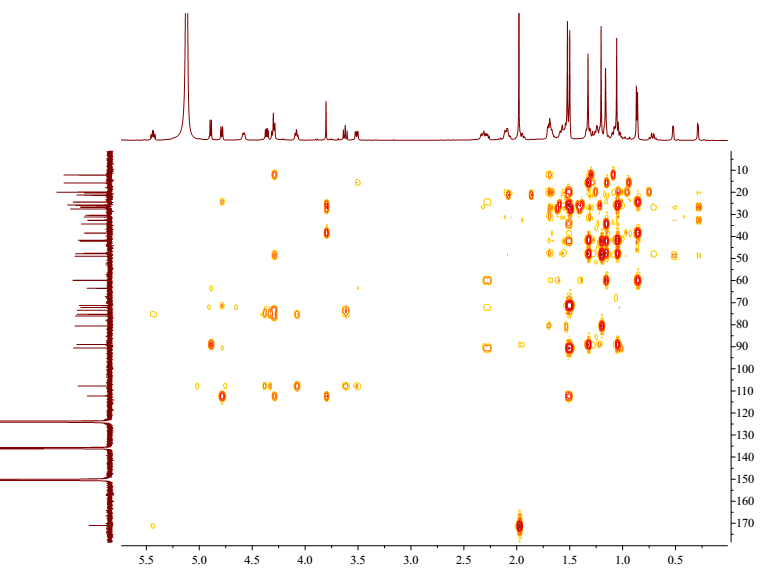


# Figure S5. COSY spectrum of compound 1 (Pyridine *d*_5_)
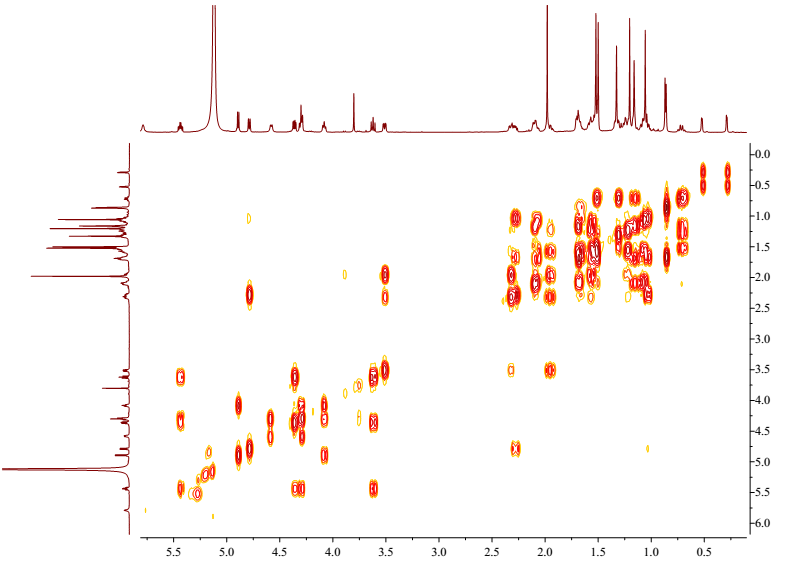


# Figure S6. ROESY spectrum of compound 1 (Pyridine *d*_5_)

**
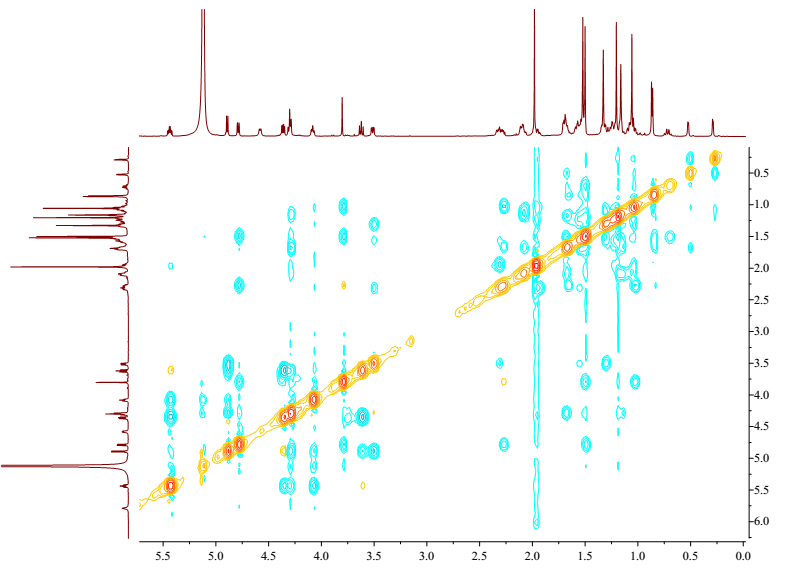
**

# Figure S7 HREIMS spectrum of compound 1
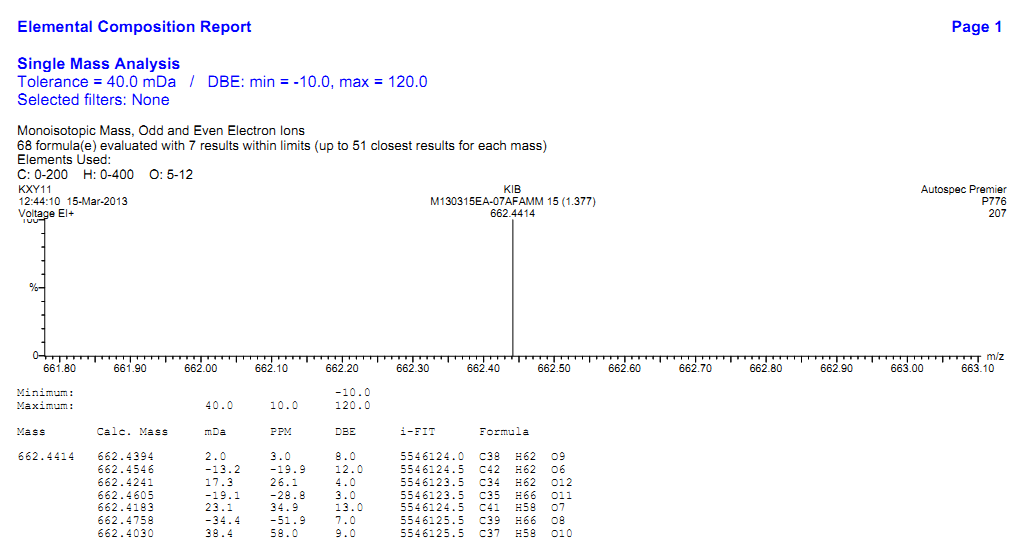


# Figure S8. ^1^H NMR spectrum of compound 2 (Pyridine *d*_5_, 600 MHz)

**
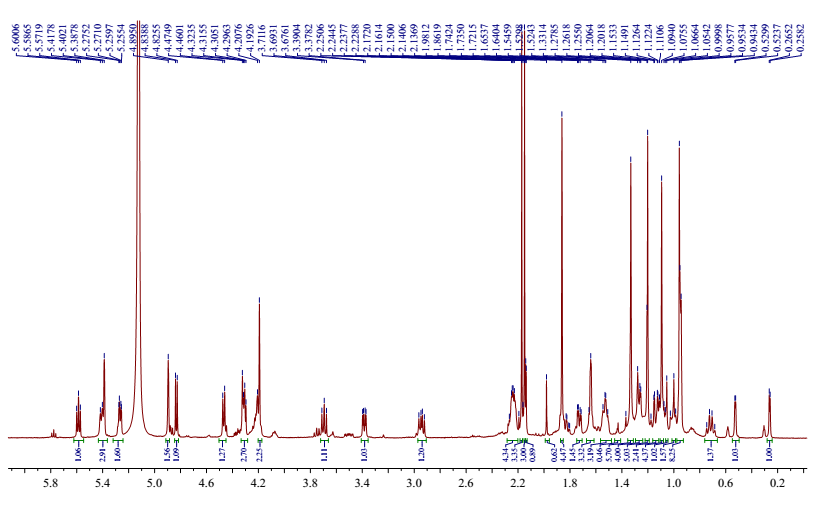
**

# Figure S9. ^13^C NMR spectrum of compound 2 (Pyridine *d*_5_, 150Hz)
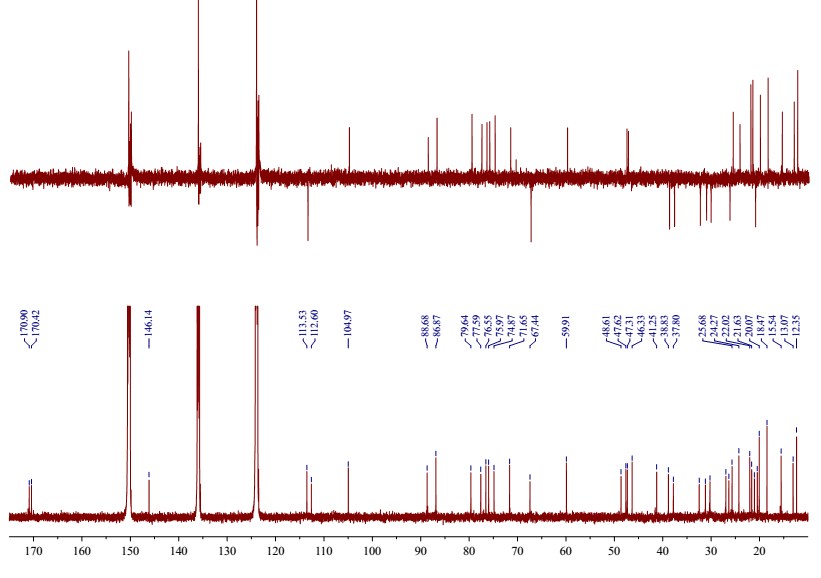


# Figure S10. HSQC spectrum of compound 2 (Pyridine *d*_5_)

**
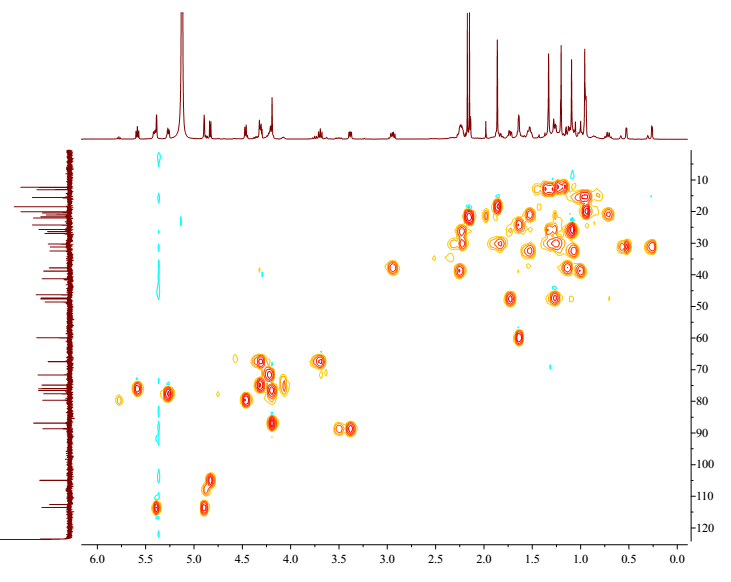
**

# Figure S11. HMBC spectrum of compound 2 (Pyridine *d*_5_)
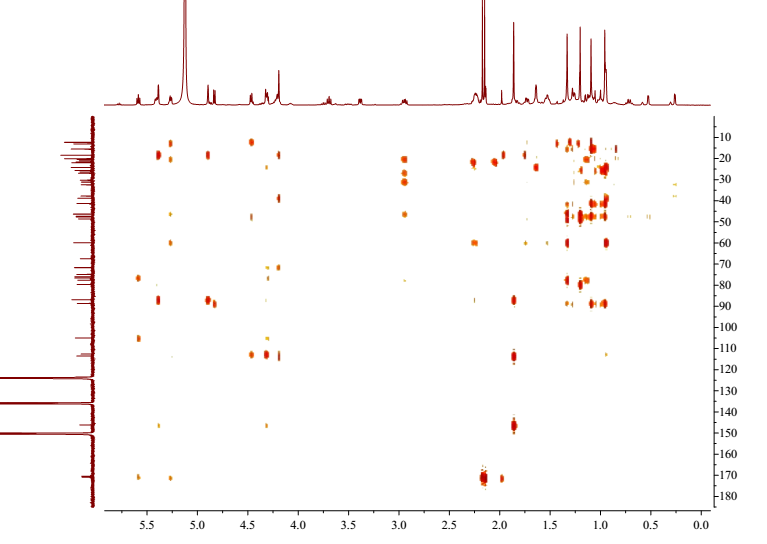


# Figure S12. COSY spectrum of compound 2 (Pyridine *d*_5_)

**
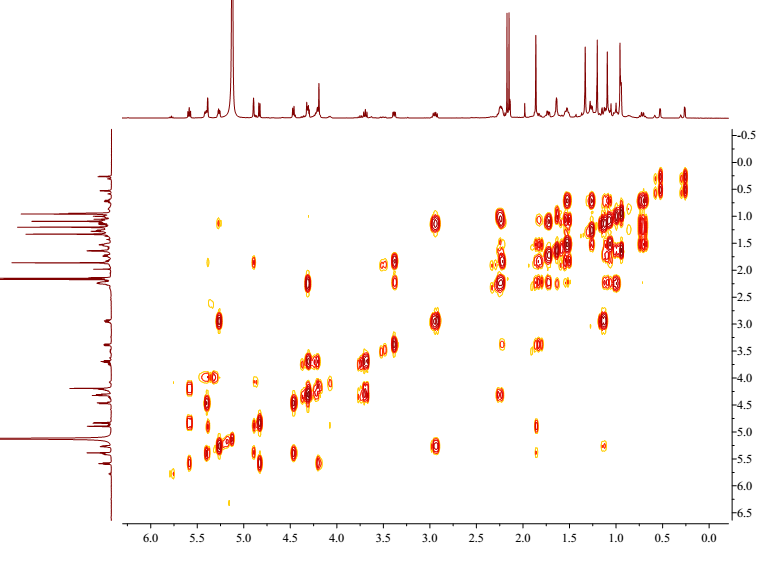
**

# Figure S13. ROESY spectrum of compound 2 (Pyridine *d*_5_)

**
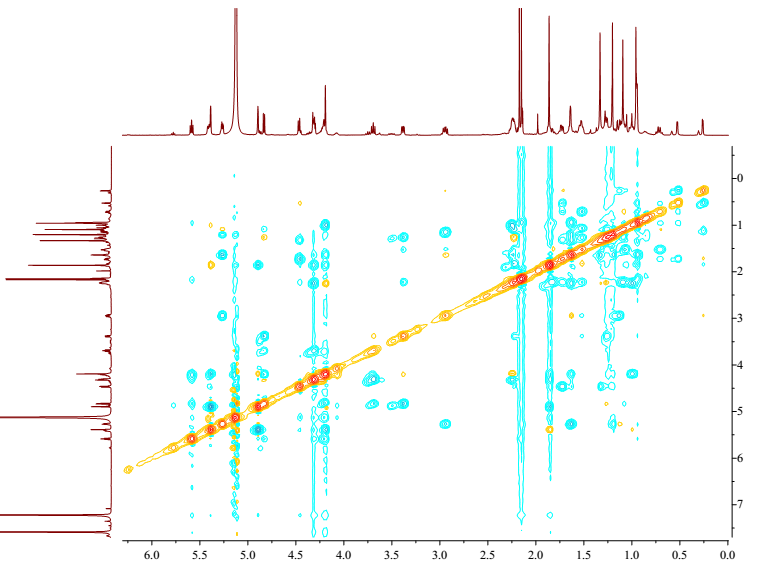
**

# Figure S14. HREIMS spectrum of compound 2


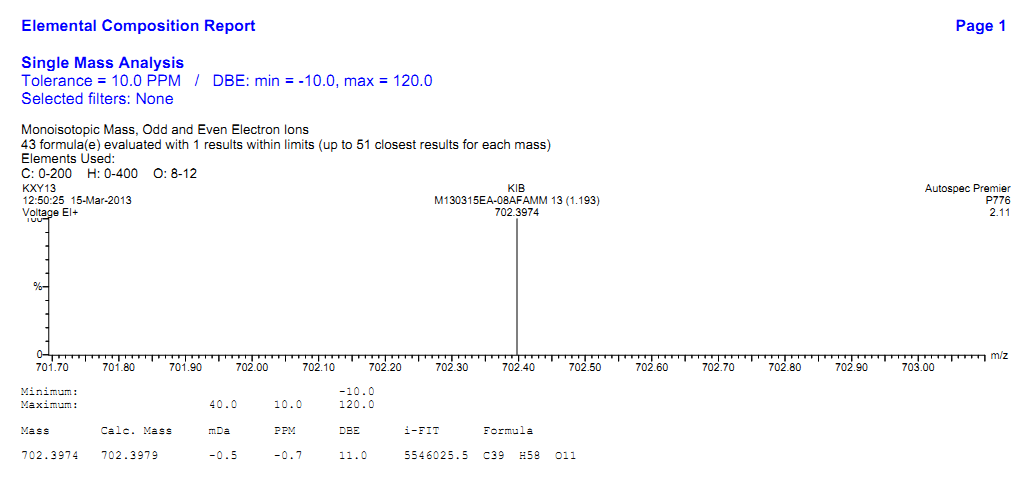


# Figure S15. ^1^H NMR spectrum of compound 3 (Pyridine *d*_5_, 600 MHz)

**
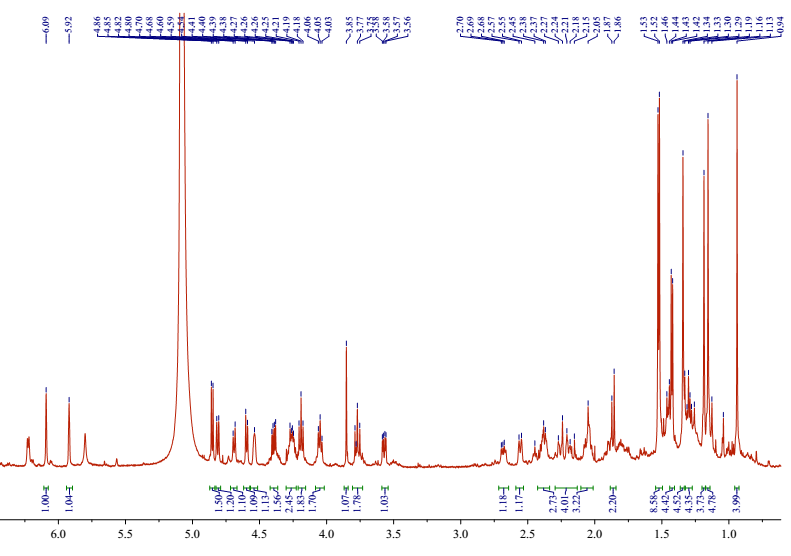
**

# Figure S16. ^13^C NMR spectrum of compound 3 (Pyridine *d*_5_, 150Hz)

**
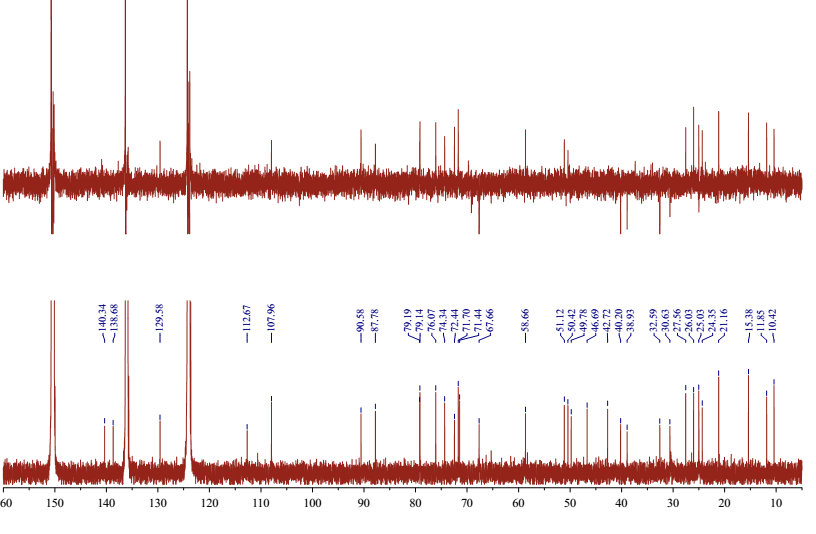
**

# Figure S17. HSQC spectrum of compound 3 (Pyridine *d*_5_)

#
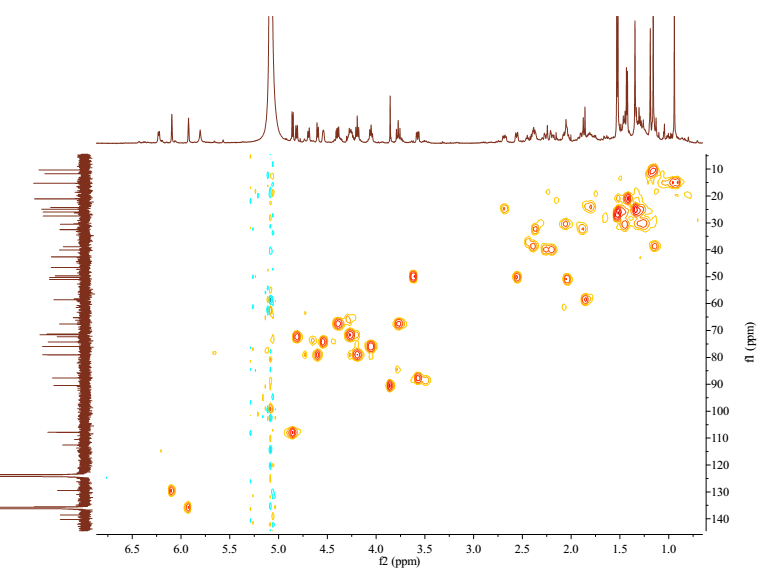


# Figure S18. HMBC spectrum of compound 3 (Pyridine *d*_5_)

**
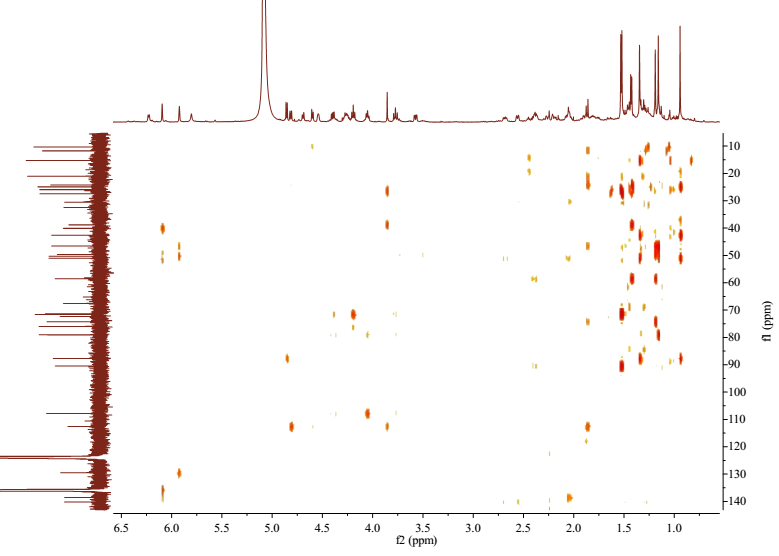
**

# Figure S19. COSY spectrum of compound 3 (Pyridine *d*_5_)
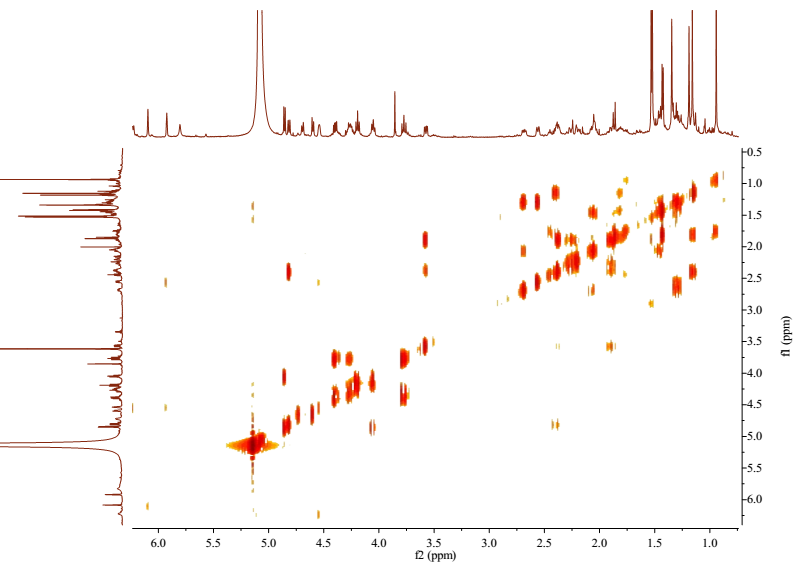


# Figure S20. HREIMS spectrum of compound 3


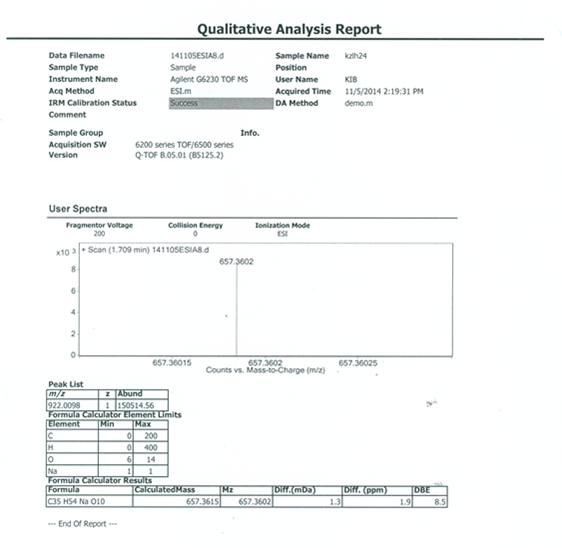


# Figure S21. ^1^H NMR spectrum of compound 4 (Pyridine *d*_5_, 600 MHz)

**
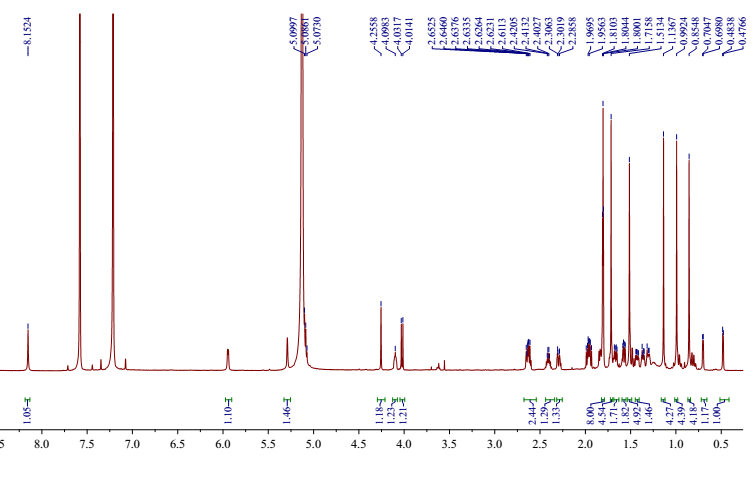
**

# Figure S22. ^13^C NMR spectrum of compound 4 (Pyridine *d*_5_, 150Hz)

**
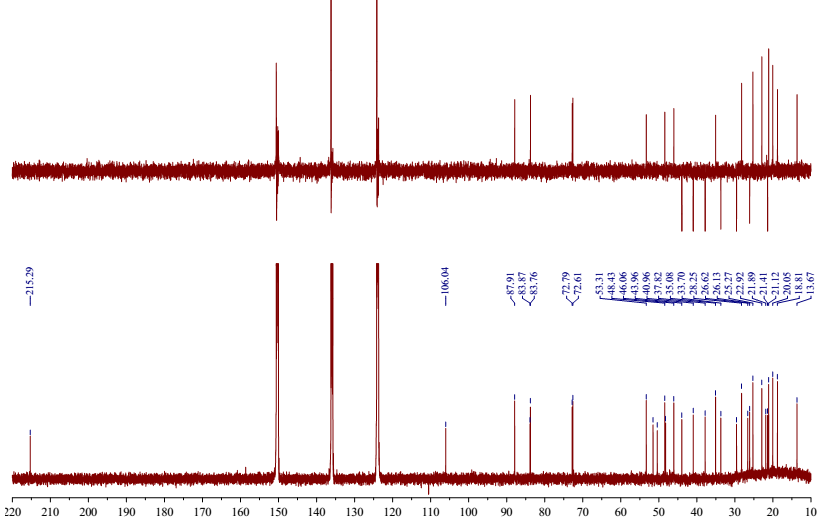
**

# Figure S23. HSQC spectrum of compound 4 (Pyridine *d*_5_)

**
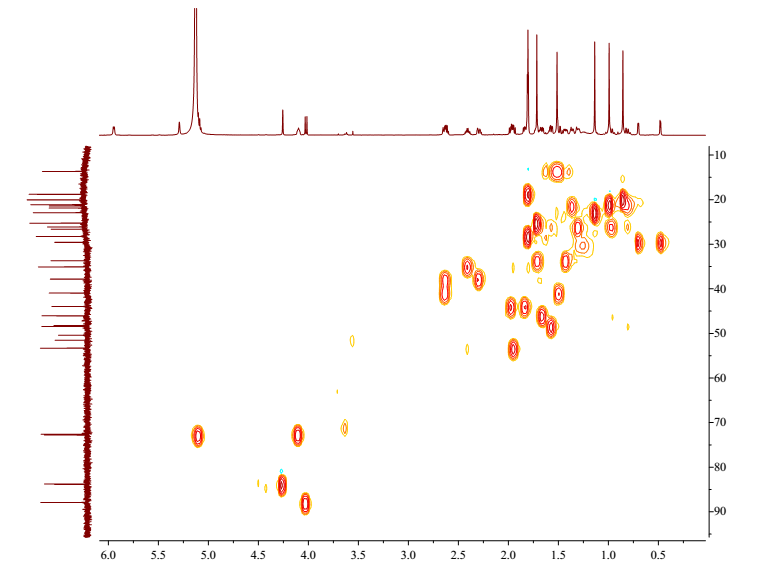
**

# Figure S24. HMBC spectrum of compound 4 (Pyridine *d*_5_)

**
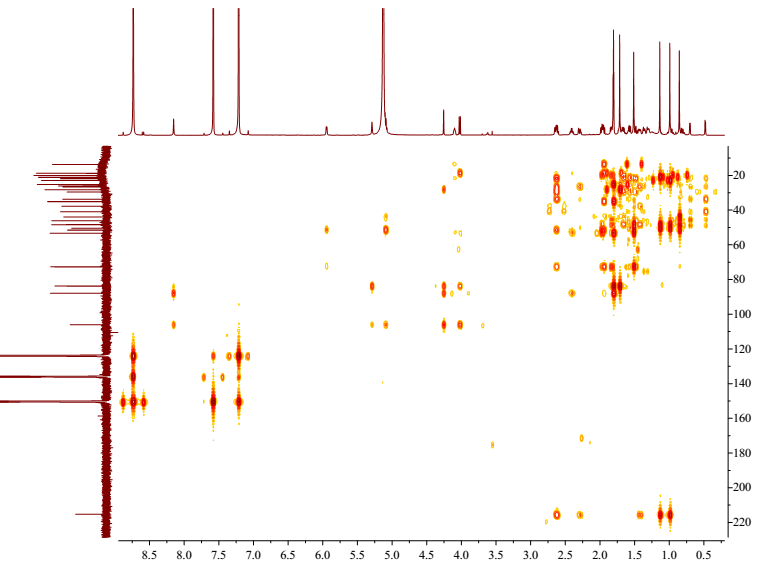
**

# Figure S25. COSY spectrum of compound 4 (Pyridine *d*_5_)

**
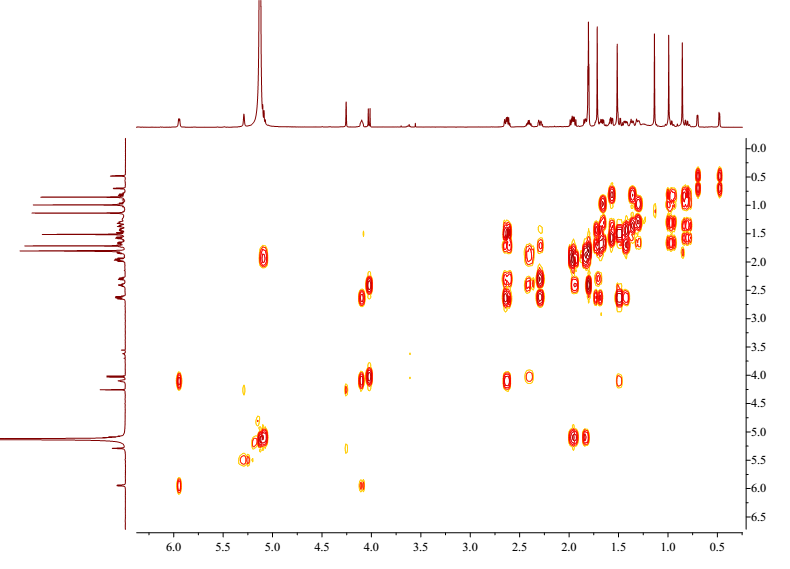
**

# Figure S26. ROESY spectrum of compound 4 (Pyridine *d*_5_)

**
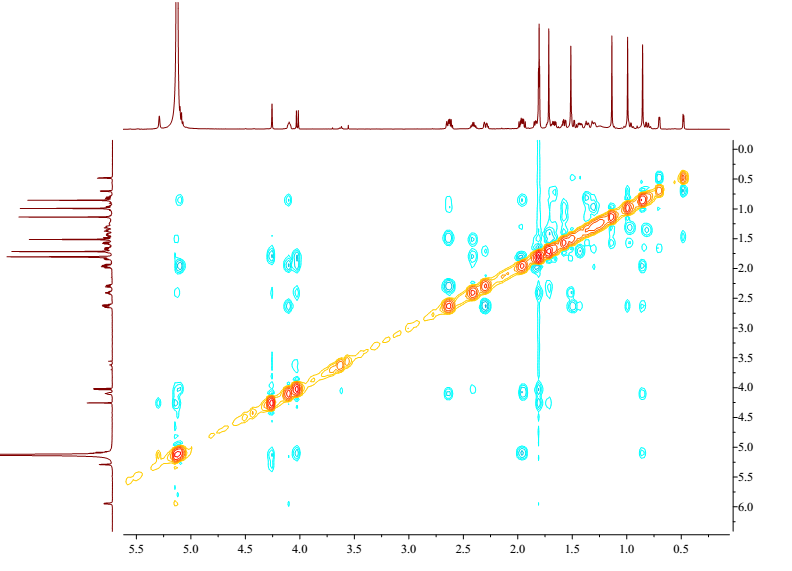
**

# Figure S27. HREIMS spectrum of compound 4


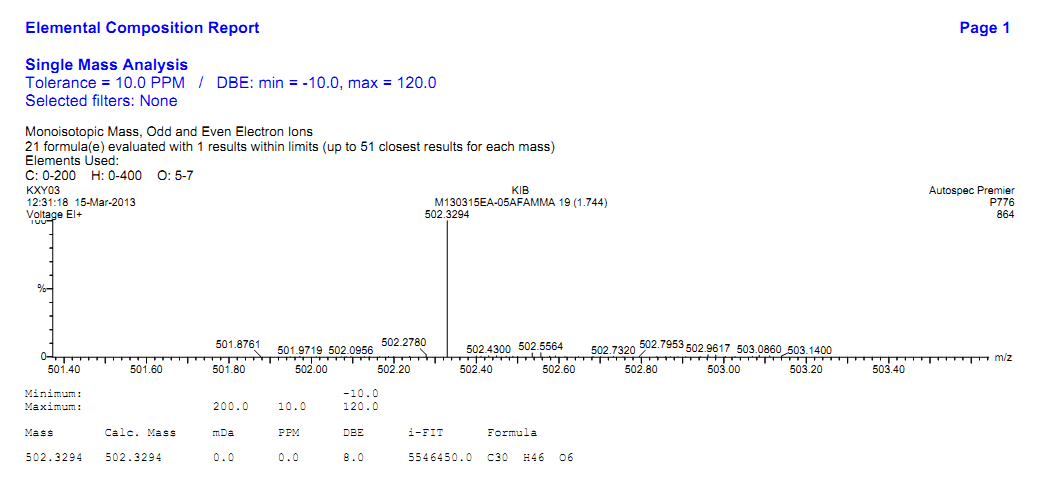


# Figure S28. ^1^H NMR spectrum of compound 5 (Pyridine *d*_5_, 600 MHz)

**
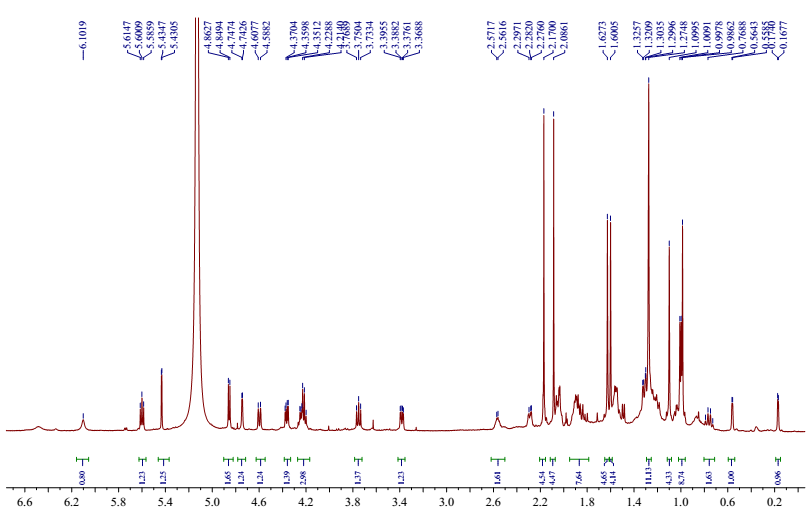
**

# Figure S29. ^13^C NMR spectrum of compound 5 (Pyridine *d*_5_, 150Hz)

**
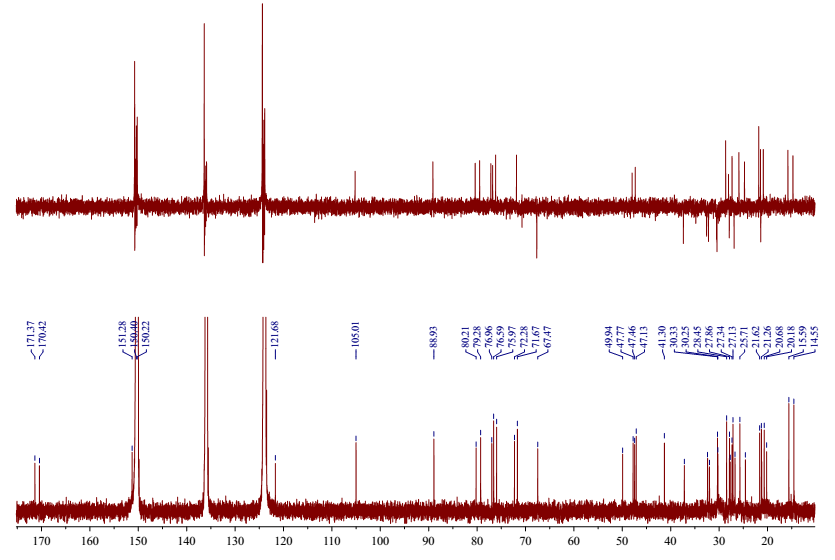
**

# Figure S30. HSQC spectrum of compound 5 (Pyridine *d*_5_)

**
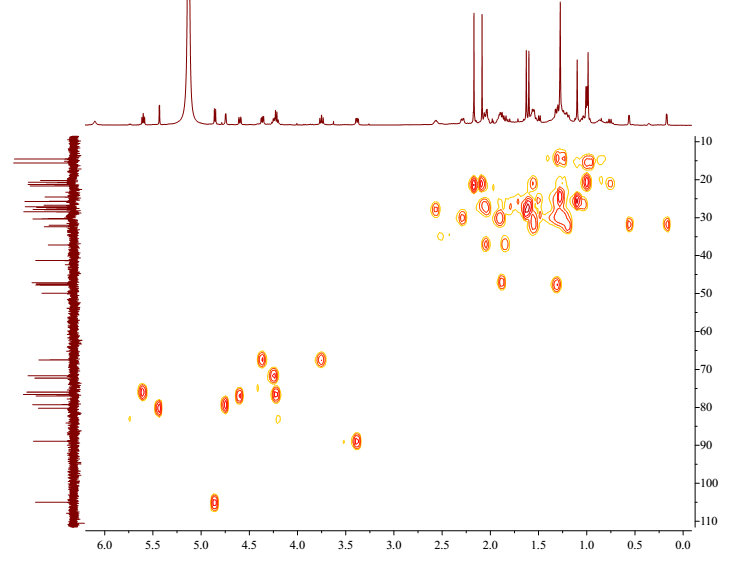
**

# Figure S31. HMBC spectrum of compound 5 (Pyridine *d*_5_)

**
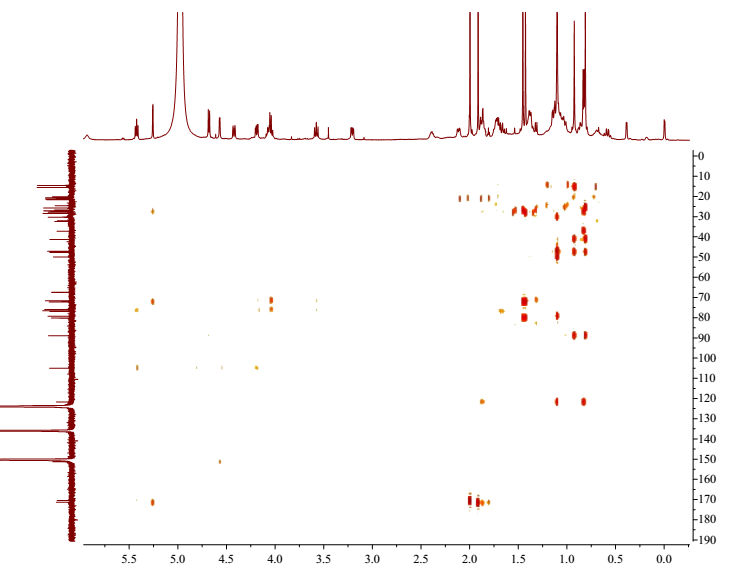
**

# Figure S32. COSY spectrum of compound 5 (Pyridine *d*_5_)

**
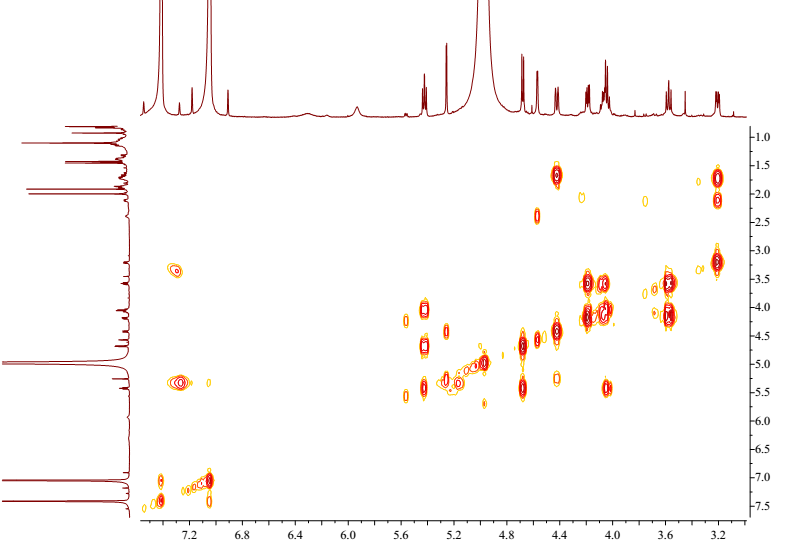
**

# Figure S33. ROESY spectrum of compound 5 (Pyridine *d*_5_)

**
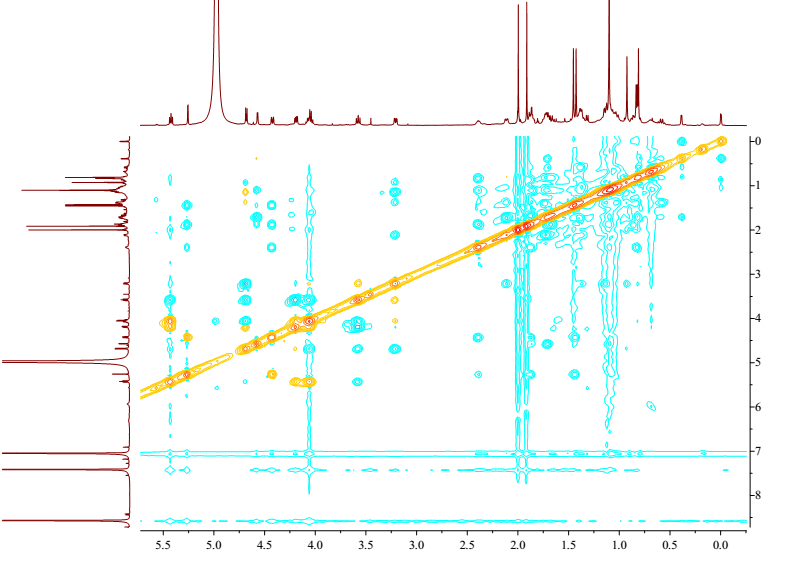
**

# Figure S34. HREIMS spectrum of compound 5


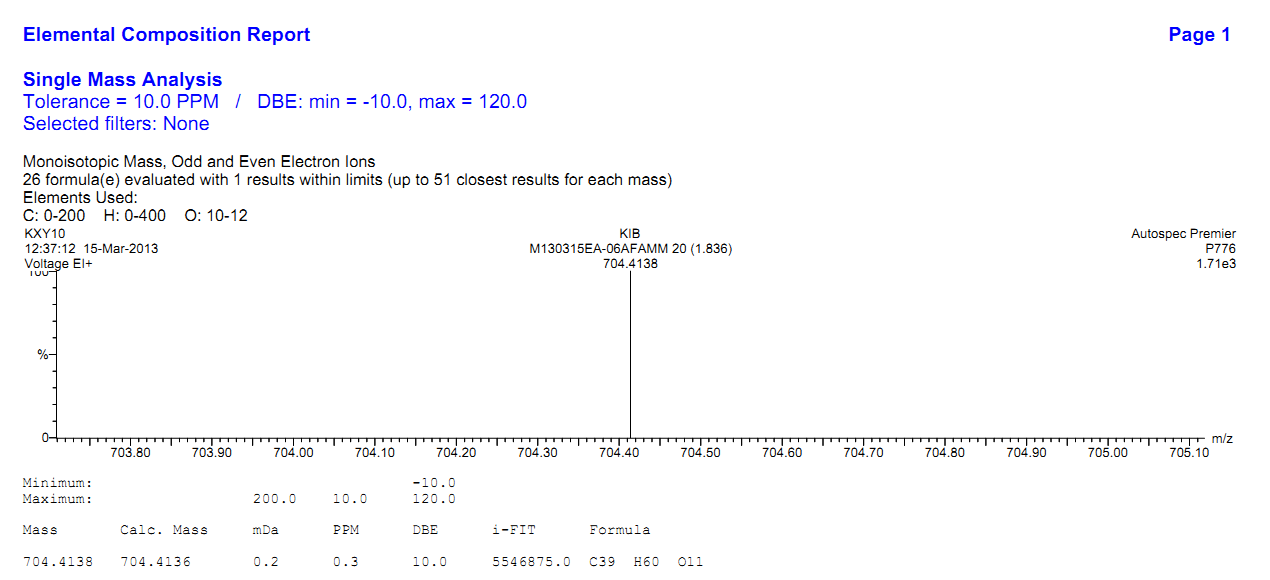


# Figure S35. ^1^H NMR spectrum of compound 6 (Pyridine *d*_5_, 600 MHz)

**
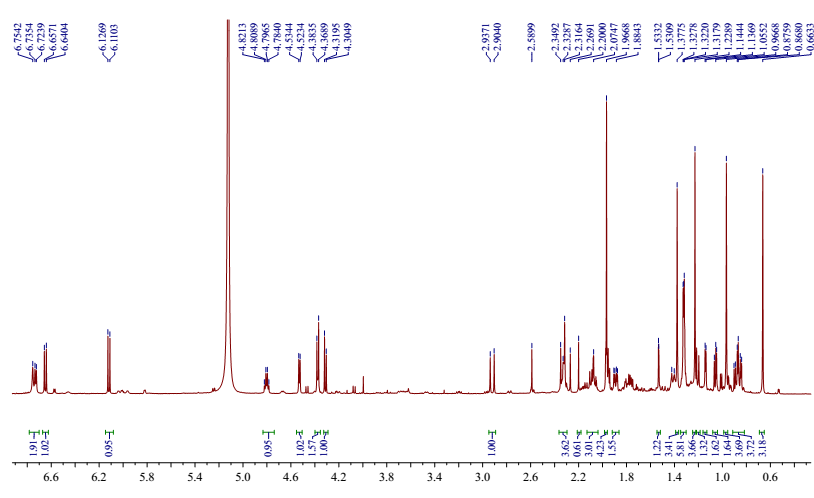
**

# Figure S36. ^13^C NMR spectrum of compound 6 (Pyridine *d*_5_, 150Hz)

**
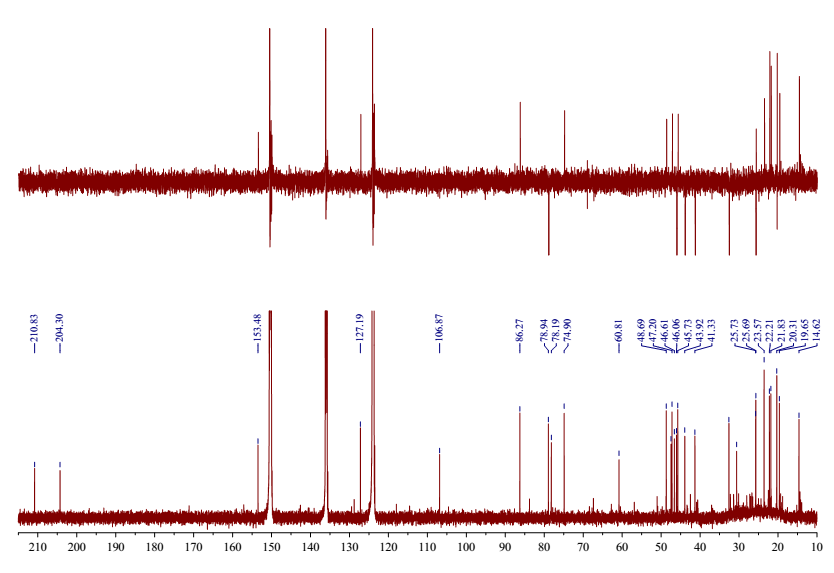
**

# Figure S37. HSQC spectrum of compound 6 (Pyridine *d*_5_)

**
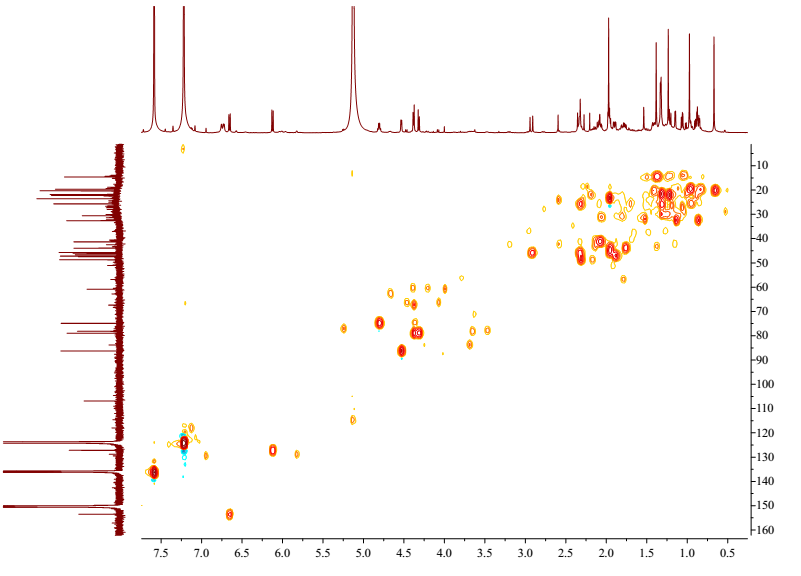
**

# Figure S38. HMBC spectrum of compound 6 (Pyridine *d_5_*)

**
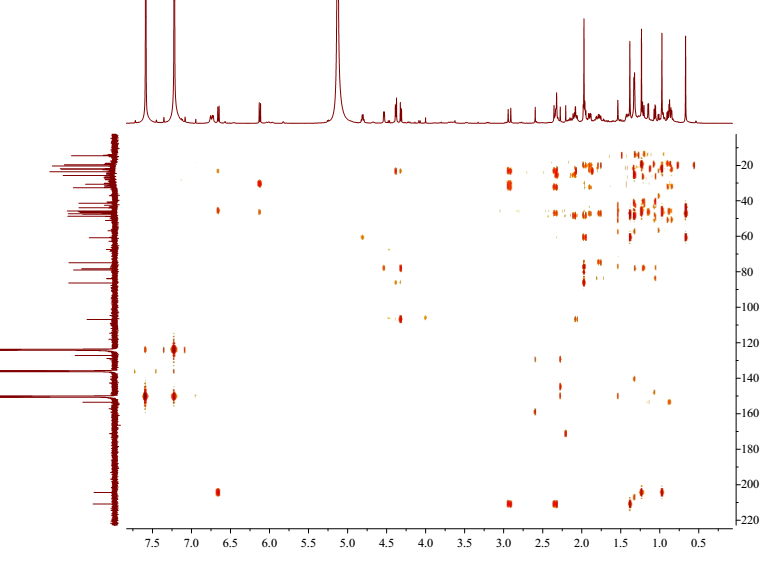
**

# Figure S39. COSY spectrum of compound 6 (Pyridine *d*_5_)

**
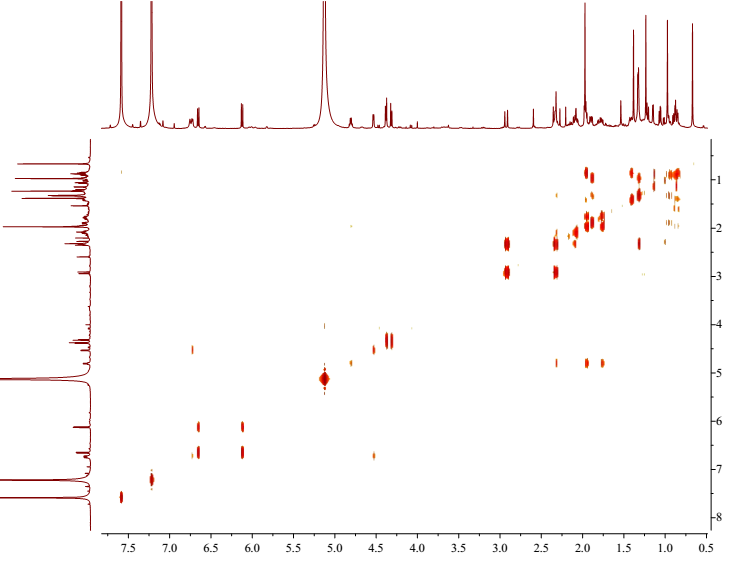
**

# Figure S40. ROESY spectrum of compound 6 (Pyridine *d*_5_)

**
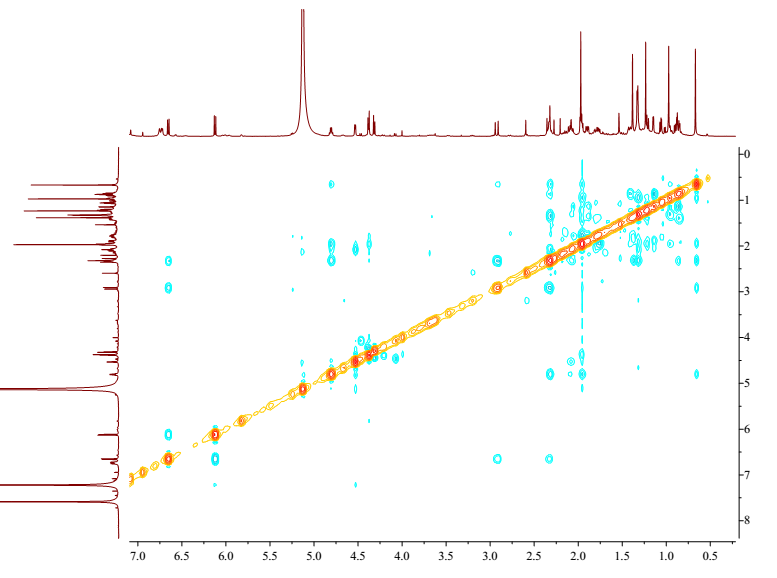
**

# Figure S41. HREIMS spectrum of compound 6


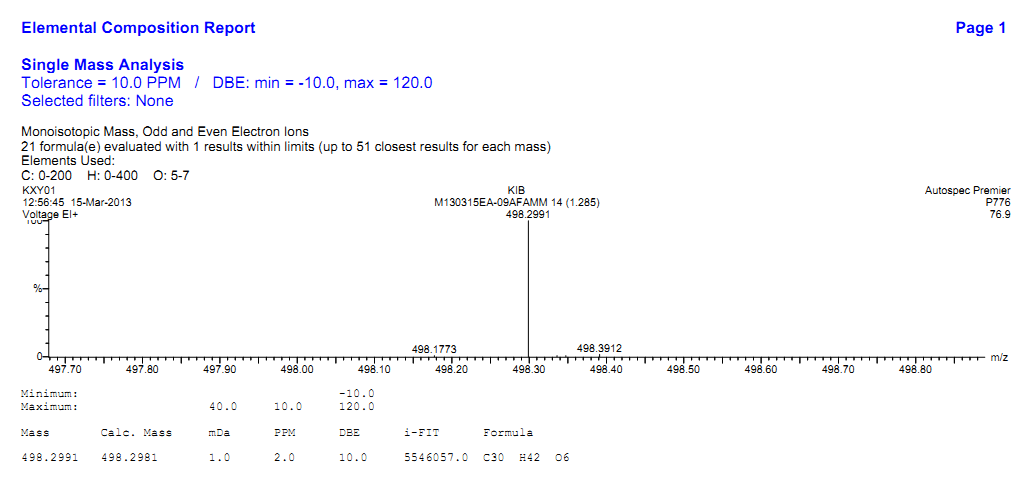

Supplement: Supplementary file 1 — Supplementary material 1 (DOC 3562 kb) [file 13659_2016_97_MOESM1_ESM.doc]
